# Syntaphilin loss enhances mitochondrial axonal transport and neuromuscular junction formation in a human stem cell derived neuromuscular assembloid model

**DOI:** 10.1186/s10020-025-01319-x

**Published:** 2025-11-05

**Authors:** Andrea Salzinger, Esra Özkan, Vidya Ramesh, Jyoti Nanda, Karen Burr, David Story, Nhan T. Pham, Siddharthan Chandran, Bhuvaneish T. Selvaraj

**Affiliations:** 1 UK Dementia Research Institute at University of Edinburgh , Edinburgh, UK; 2 Centre for Clinical Brain Sciences and Euan McDonald Centre for MND Research, University of Edinburgh, Edinburgh, UK; 3 Institute for Regeneration and Repair, University of Edinburgh, Edinburgh, UK

**Keywords:** Assembloid, Stem cells, Organoid, Mitochondria, Axonal transport, Neuromuscular junction, Neuromuscular diseases

## Abstract

**Background:**

The neuromuscular junction (NMJ) is the synapse between motor neurons and skeletal muscle and controlls movement. Impaired synaptic transmission and NMJ degeneration has been observed during healthy ageing and is also implicated in several neuromuscular diseases. On account of the high energy demands of being distally located and large sized, NMJs are enriched with mitochondria. This enrichment is dependent on transport of mitochondria across the axon to the NMJ.

**Methods:**

We first established a human 3D neuromuscular assembloid model to study in-vitro NMJs, by fusing human stem cell derived spinal cord organoids and primary skeletal muscle spheroids. To determine whether enhancing axonal mitochondrial transport modulates NMJ formation and maintenance, we generated a CRISPR-Cas9 meditated knockout of syntaphilin in human stem cells.

**Results:**

Firstly, we characterised the neuromuscular assembloid model which showed functional innervated NMJs as measured by juxtaposed neurofilament^+^ axons and α-bungarotoxin^+^ acetylcholine receptors. Secondly, we showed that spinal cord selective genetic ablation of syntaphilin – an axonally localised mitochondrial anchor protein – resulted in increased mitochondrial motility in motor neurons, and consequently increased axonal density and NMJ formation.

**Conclusion:**

This proof-of-concept study demonstrated that enhancing mitochondrial mobility could provide a therapeutic target to prevent NMJ degeneration.

**Supplementary Information:**

The online version contains supplementary material available at 10.1186/s10020-025-01319-x.

## Introduction

The neuromuscular junction (NMJ) is one of the largest and best-studied synapses in the human nervous system and undergoes dynamic remodelling during development (Sanes and Lichtman 1999; Rodríguez Cruz et al. 2020; Burden et al. 2018; Pun et al. 2006). It connects the endplates of spinal cord motor neurons (MN) with skeletal muscle (SkM) and is responsible for the control of voluntary muscle contraction. Physiologically, the pre-synaptic cholinergic MN releases acetylcholine (ACh) into the synaptic cleft for post-synaptic uptake via nicotinic acetylcholine receptors (AChRs) at the SkM. Upon depolarization of the post-synaptic membrane, calcium is released from the sarcoplasmic reticulum, leading to muscle contraction (Rodríguez Cruz et al. 2020). NMJ dysfunction, for instance impaired neuromuscular transmission or NMJ degeneration, has been described in several neuromuscular disorders (Rodríguez Cruz et al. 2020)—neuromuscular autoimmune diseases (Lipka and Verschuuren 2024; Tannemaat et al. 2024), spinal cord injury (Burns et al. 2007; Ollivier-Lanvin et al. 2009; Mantilla et al. 2007) and in neurodegenerative disorders such as amyotrophic lateral sclerosis (ALS) and spinal muscular atrophy (Verma et al. 2022; Dadon-Nachum et al. 2011; Boido and Vercelli 2016; Fulceri et al. 2021).

Until recently, NMJ studies have largely relied on in-vivo animal models. However, the finding of important interspecies differences between human and rodent NMJs at both morphological and molecular level has led to the development of complementary human based models (Jones et al. 2017; Slater 2017). Advances in stem cell technology have enabled the generation of diverse human in-vitro NMJ models, including bioengineered and 3D organoid models, each offering distinct advantages (Pereira et al. 2021; Stoklund Dittlau et al. 2021); Stoklund Dittlau et al. 2023; Massih et al. 2023; Santhanam et al. 2018; Vila et al. 2021; Faustino Martins et al. 2020; Urzi et al. 2023; Andersen et al. 2020; Nebol and Gouti 2024; Guo et al. 2020). Compartmentalised models employing microfluidic devices offer a controlled environment to study neuron-muscle crosstalk, with the option of adding additional cell types (Stoklund Dittlau et al. 2021; Stoklund Dittlau et al. 2023), and are specifically advantageous for high throughput studies and functional assays, including optogenetic manipulations (Pereira et al. 2021); Stoklund Dittlau. 2021; Stoklund Dittlau et al. 2023; Massih et al. 2023; Santhanam et al. 2018; Vila et al. 2021). Meanwhile, 3D models offer a mixed cellular population of progenitor and glial cells in addition to neurons and skeletal muscle, as well as the possibility of long-term cultures, often up to several months (Massih et al. 2023; Pereira et al. 2021; Faustino Martins et al. 2020; Urzi et al. 2023; Andersen et al. 2020). In summary, human in-vitro NMJ models provide versatile platforms to study NMJ formation, functionality and pathology (Pereira et al. 2021; Stoklund Dittlau et al. 2021; Stoklund Dittlau et al. 2023; Massih et al. 2023; Santhanam et al. 2018; Vila et al. 2021; Faustino Martins et al. 2020; Urzi et al. 2023; Andersen et al. 2020; Nebol and Gouti 2024; Guo et al. 2020).

Mitochondria are transported, amongst others to the NMJ, through fast-axonal transport mechanisms (Mandal and Drerup 2019; Saxton and Hollenbeck 2012). Given the distal location of the NMJ (Hagemann et al. 2022), maintaining mitochondrial homeostasis is particularly crucial to facilitate, for instance, local energy production (Anagnostou and Hepple 2020; Altman and Perlson 2021; Altman et al. 2019). NMJ mitochondrial dysfunction has been implicated in disruption of NMJ maintenance and was found in a range of disorders (Anagnostou and Hepple 2020; Altman and Perlson 2021; Altman et al. 2019). In ALS, where NMJ degeneration is an early pathology, both intrinsic mitochondrial function as well as mitochondrial axonal transport are disrupted in MNs (Salam et al. 2021; Wang et al. 2021; Singh et al. 2021; Mehta et al. 2021; Kodavati et al. 2020). However, it is not known whether mitochondrial axonal transport in MNs regulate NMJ maintenance and function (Fulceri et al. 2021; Hayes et al. 2019; Ke et al. 2021; Han et al. 2020).

Syntaphilin (SNPH) is an axonal localised mitochondrial anchor protein, which functionally inhibits mitochondrial transport by immobilising mitochondria to the microtubule (Kang et al. 2008). Syntaphilin plays a crucial role in neuronal homeostasis both in health and disease. Upon mitochondrial stress, SNPH bound mitochondria are released and retrograde transport of damaged mitochondria is promoted to maintain neuronal health (Lin et al. 2017). Reduction of SNPH results in increased mobility of axonal mitochondria and enhanced short-term facilitation of hippocampal neurons (Kang et al. 2008). Inhibition of SNPH has also been shown to promote axonal regeneration after peripheral nerve crush injury (Zhou et al. 2016) and spinal cord injury (Wu et al. 2023). SNPH has further been implicated in ALS by evidence of interaction with FUS – an ALS linked protein (Salam et al. 2021). Notwithstanding these studies, the impact of SNPH inhibition and subsequent axonal mitochondrial transport on NMJ maintenance remains to be studied.

Against this background, we used a human 3D in-vitro assembloid model of functional innervated NMJs to examine the impact of selective genetic ablation of syntaphilin on mitochondrial motility, axonal length and NMJ innervation. Selective depletion of SNPH in spinal cord organoids led to increased mitochondrial mobility, axonal density and enhanced NMJ innervation.

## Results

### Generation and characterization of a human stem cell derived 3D model of the neuromuscular junction

**Fig. 1:**
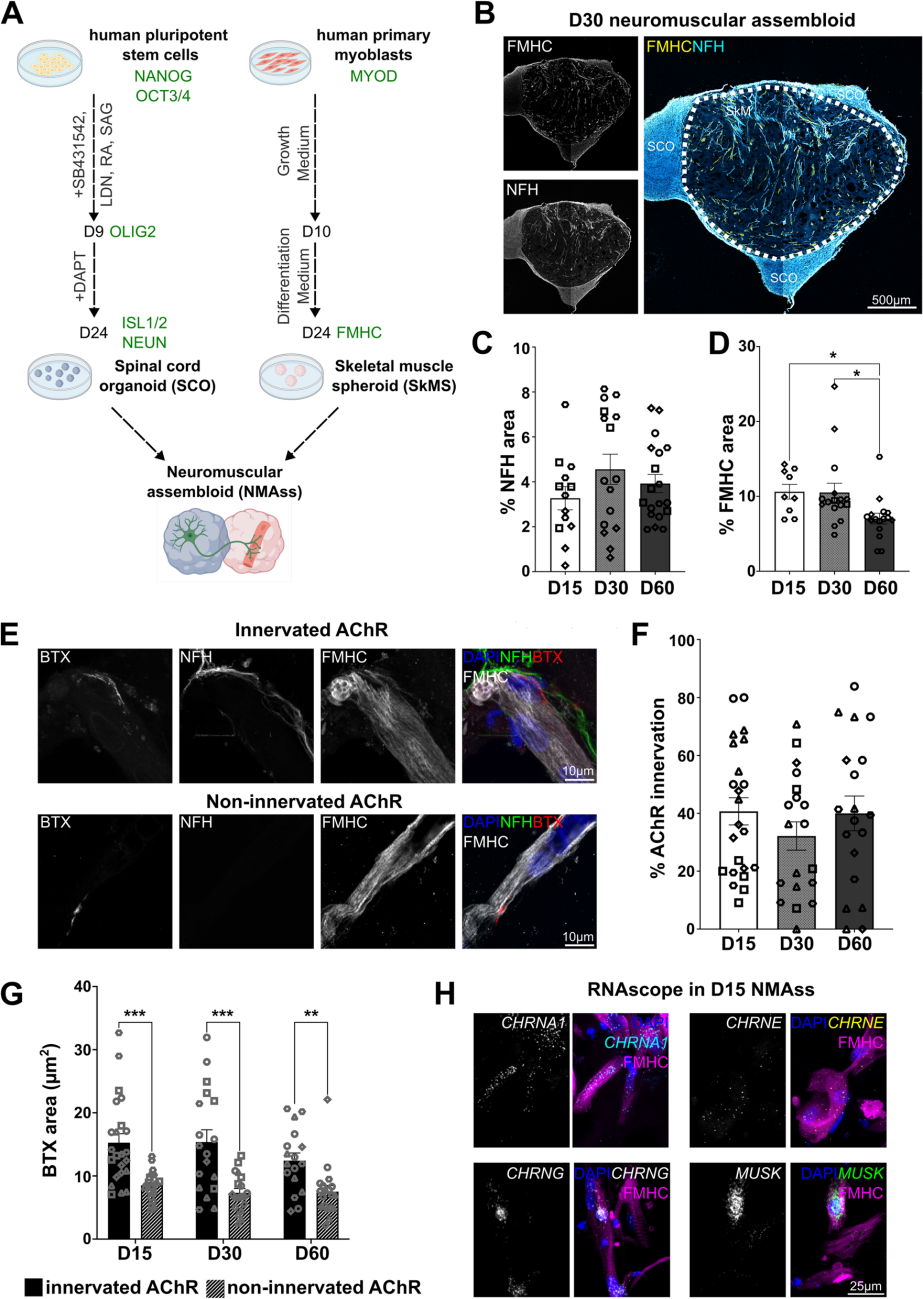
Generation and characterization of the neuromuscular assembloid. **A** Schematic of neuromuscular assembloid (NMAss) generation. Human pluripotent stem cells were differentiated to spinal cord organoids (SCO) for 24 days using dual SMAD inhibition (SB, LDN) for neuroectoderm patterning and retinoic acid (RA), smoothened agonist (SAG) for anterior–posterior and dorsal–ventral axis development, respectively. Primary skeletal myoblasts were differentiated into skeletal muscle spheroids (SkMS). After 24 days, SCO and SkMS were assembled into neuromuscular assembloids. Created with biorender. **B** Axonal density of SCO into SkMS in D30 NMAss. Scale bar: 500 µm. **C** Quantification of axonal density in SkMS as percentage of NFH area within SkMS showed no significant difference over time. Kruskal–Wallis with Dunn’s correction, p = ns, N = 3–4, n = 13–19, mean D15 = 3.2%, D30 = 4.5%, D60 = 3.9%. **D** Quantification of FMHC area within SkMS showed a significant decline of muscle area at D60 after assembly. Kruskal–Wallis with Dunn’s correction, p = 0.0132 (D15-D60), p = 0.0270 (D30-D60), N = 2–4, n = 9–16, mean D15 = 10%, D30 = 10%, D60 = 7%. **E** Example image of in-vitro NMJ showing co-localization of AChRs, labelled by BTX, at post-synapse and NFH ^+^ pre-synaptic axon. Example image of BTX ^+^ AChR cluster without NFH co-localization. Scale bars: 10 µm. **F** Formation of NFH ^+^/BTX ^+^ in-vitro NMJs was not significantly altered over time. Brown-Forsythe and Welch’s one-way ANOVA with Dunnet’s T3 multiple comparison test, p = ns, *N* = 4–5, n = 19–23, mean D15 = 40%, D30 = 32%, D60 = 40%. **G** Quantification of post-synaptic BTX area showed no significant difference between assessed timepoints but showed a significant difference between the NFH positive AChR cluster and NFH negative AChR cluster. Two-way ANOVA with Šídák's multiple comparisons test, p = 0.0001(D15), p = 0.0008(D30), p = 0.0033(D60), *N* = 4–5, *n* = 17–23, mean NFH ^+^/BTX ^+^ AChR cluster D15 = 15µm^2^, D30 = 15µm^2^, D60 = 12.4µm^2^, mean NFH^ −^/BTX^ +^ AChR cluster D15 = 8.5µm^2^, D30 = 7.3µm^2^, D60 = 7.5µm^2^. Each data point represents one NMAss. Different shapes represent different N. Data presented as mean ± SEM. **H** RNA expression of α1 (CHRNA1), ε (CHRNE) and γ (CHRNG) subunits of AChR, as well as MUSK in D15 neuromuscular assembloid alongside with FMHC protein staining. Scale bars: 25 µm

To study human in-vitro NMJs, we generated a 3D neuromuscular assembloid model, adapted from Andersen et al. 2020*,* by fusing human pluripotent stem cell (hPSC) derived spinal cord organoids (SCOs) (Mehta et al. 2022; Maury et al. 2015; Selvaraj et al. 2018) with human primary skeletal muscle spheroids (SkMS, Fig. 1A) at day 24 post differentiation. The MN fate of stem cell derived SCOs was confirmed by expression of OLIG2 at D9 (Supplementary Fig. 1 A) and ISL1/2, NEUN at D24 (Supplementary Fig. 1B). A minor percentage of cells in D24 SCOs was positive for GABA, but negative for PV (Supplementary Fig.1 C). Neither D9, nor D24 SCOs were positive for S100B (Supplementary Fig. 1D), while we observed SOX9^+^ astrocyte progenitors in D24 and D56 SCOs (Supplementary Fig. 1E). GFAP^+^ astrocytes were only found in D56 SCOs (Supplementary Fig. 1E). No MBP^+^ oligodendrocytes were identified (Supplementary Fig. 1 F). Human primary SkMS contained a heterogenous population of mature and differentiating skeletal muscle cells, confirmed by expression of MYOD (myoblast progenitor), TITIN (intermediate filament marker) and FMHC – a marker for fast fatigable skeletal muscle fibres and the muscle cell type primarily affected in ALS (Mukund and Subramaniam 2020; Schweingruber and Hedlund 2022) (Supplementary Fig. 1G, G’).

Differentiated SCOs and SkMS were fused at day 24 to generate neuromuscular assembloids (NMAss, Fig. 1A). NFH^+^ motor axons projected from the SCO into SkMS which was assessed temporally at D15, D30 and D60 (Fig. 1B) and revealed no significant change (Fig. 1B, C). The area occupied by FMHC^+^ skeletal muscle fibres, was moderately reduced over the time period studied (Fig. 1B, D). NMAss, assessed at day 30, consist of ISL1/2^+^, NEUN^+^ and NFH^+^ MNs (Supplementary Fig. 2 A, Fig. 1B), SOX9^+^, GFAP^+^ astrocytes (Supplementary Fig. 2B), MBP^+^ oligodendrocytes (Supplementary Fig. 2 C) and DESMIN^+^, FMHC^+^ and TITIN^+^ muscle fibres (Supplementary Fig. 2D, Fig. 1B). Interestingly, oligodendrocytes identified by MBP were observed only in NMAss and not in SCOs (Supplementary Fig. 1 F). In conclusion, the cellular composition of NMAss is comparable to SCO and SkMS cultures in isolation, with the exception of MBP which was only present in NMAss.

### NMAss form functional in-vitro neuromuscular junctions which express key molecules of the AChR clustering pathway

To investigate the formation of in-vitro NMJs in NMAss, we performed immunofluorescent cytochemistry for post-synaptic acetylcholine receptors (AChR) using bungarotoxin (BTX) and neurofilament heavy chain (NFH) for pre-synaptic motor neuron axons. We observed 1) BTX^+^ AChR clusters co-localising with NFH, which we henceforth refer to as innervated AChR or in-vitro NMJs and 2) BTX^+^ AChR clusters without NFH which we refer to as non-innervated AChR (Fig. 1E). To confirm that NFH^+^/BTX^+^ AChR clusters indeed represent a synapse, we performed co-localization studies with the synaptic marker synaptic vesicle glycoprotein 2 A (SV2A). We identified that 94% of NFH^+^/BTX^+^ innervated AChRs co-localised with the presynaptic protein SV2A, while the majority of non-innervated (NFH^−^/BTX^+^) AChR were negative for SV2A (Supplementary Fig. 3 A, B). This strongly suggest that NFH^+^/BTX^+^ ACHRs are predominantly colocalised with presynaptic SV2A and both NFH and SV2A can be used as a pre-synaptic marker for the classification of AChR innervation (Supplementary Fig. 3 A, B).

NMJ formation and morphology were assessed in a temporal manner at D15, D30 and D60 (Fig. 1E-G). At D15, 40% of AChR within NMAss were innervated by MN axons which did not significantly change over time (Fig. 1F). The post-synaptic BTX^+^ area of in-vitro NMJs was significantly larger than the post-synaptic area of NFH negative AChR cluster across all three time points (Fig. 1G). To determine if formation of BTX^+^ post-synaptic AChR is only observed when in contact with MNs, SkMS were analysed for the presence of AChR cluster. Although highly variable across multiple batches, SkMS were occasionally able to form BTX^+^ AChR (Supplementary Fig. 3 C), and their size was similar to NFH negative AChR within NMAss (Supplementary Fig. 3D). Given the absence of MNs, BTX^+^ AChR in SkMS were not innervated (Supplementary Fig. 3E). Spontaneous contractions within NMAss as well as spontaneous calcium waves within muscle fibres of NMAss were also observed (Supplementary Video 1–4). Addition of curare, an inhibitor of the AChRs, stopped spontaneous contractions (Supplementary Video 5). No spontaneous contractions were observed in SkMS (Supplementary Video 6). Taken together, these findings are consistent with the formation of functional NMJs.

Next, we analysed mRNA expression of genes essential for AChR clustering. In specific, we studied AChR subunits, α1, ε and γ, as well as the muscle specific kinase (MuSK) using RNAscope in D15 (Fig. 1H), D30 (Supplementary Fig. 4 A) and D60 NMAss (Supplementary Fig. 4B). RNAscope probes were assessed for specificity using appropriate negative controls such as a probe against bacterial gene *DapB*, no probe and no secondary controls (Supplementary Fig. 4 C). *CHRNA1*, the α1 subunit of AChR was widely expressed and spread throughout the muscle fibres. *CHRNG*, the γ subunit of AChR, also known as the embryonic subunit, was clustered at individual nuclei of muscle fibres (Cetin et al. 2020) (Fig. 1H). *CHRNE*, the ε subunit of AChR, and adult subunit, was modestly expressed. This suggests that the AChR subunit switch from γ subunit to ε subunit (embryonic to adult), which typically occurs postnatally (Cetin et al. 2020), is limited at the evaluated time-points (Fig. 1H). Lastly, *MUSK*, a vital protein required for AChR clustering, is highly expressed in D15 NMAss and mainly found as cluster within nuclei (Fig. 1H). Temporal assessment of RNA expression of *CHRNA1*, *CHRNG*, *CHRNE* and *MUSK* showed comparable expression patterns across the analysed timepoints (Fig. 1H, Supplemental Fig. 4 A, B). To conclude, the NMAss model recapitulates important morphological, molecular and functional aspects of known NMJ development.

### SNPH loss leads to enhanced mitochondrial mobility in MN axons

**Fig. 2:**
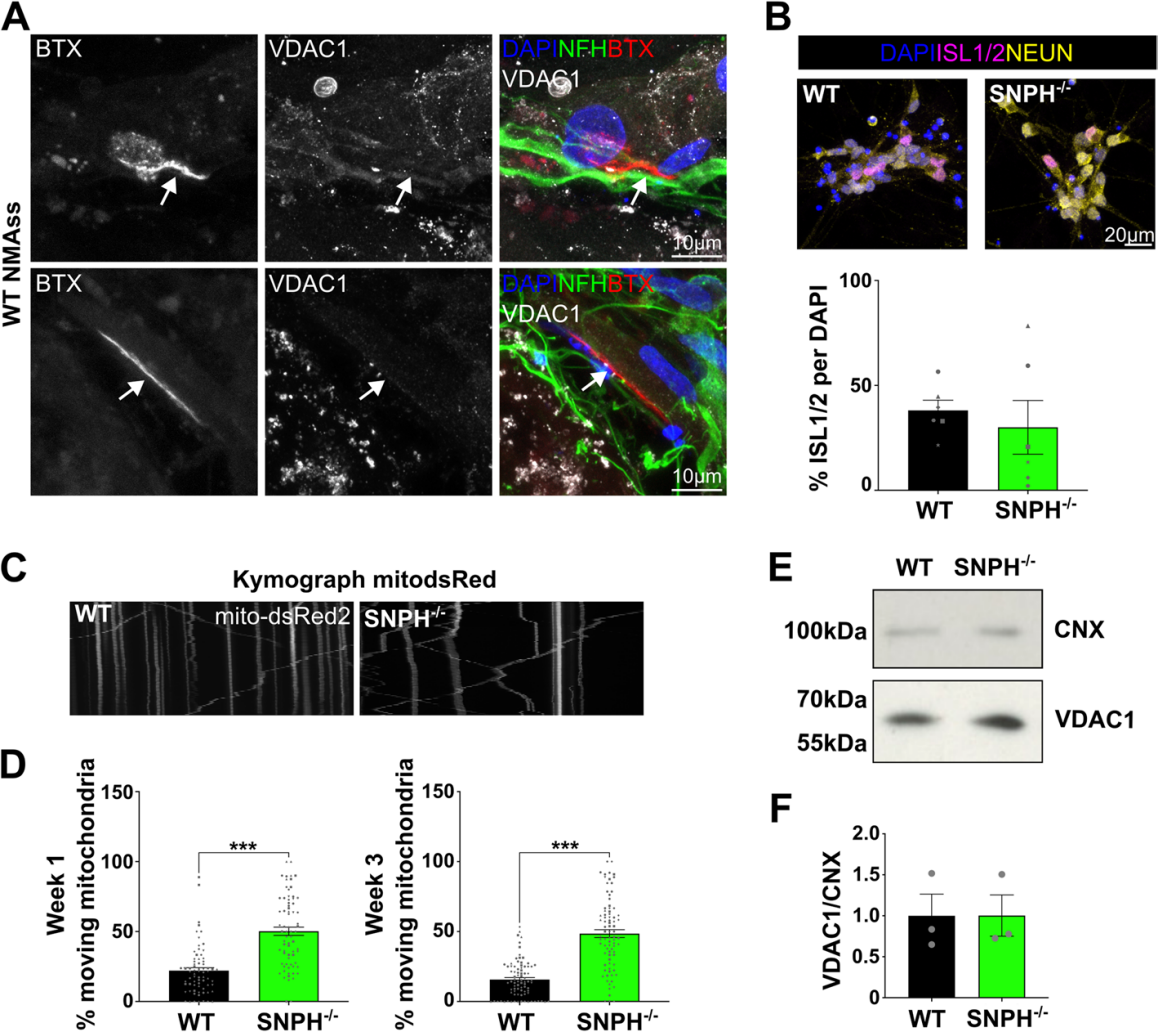
Loss of SNPH promoted increased mitochondrial mobility in motor neuron axons. **A** Presence of VDAC1 ^+^ mitochondria in proximity of the NMJ within NMAss. Scale bar: 10 µm. **B** Representative images of one-week 2D MN cultures and ISL1/2 quantification. DAPI: nuclei, ISL1/2: motor neuron marker, NEUN: pan-neuronal marker. Scale bar: 20 µm. Mean WT = 38%, mean SNPH ^−/−^ = 30%. Welch’s t-test, p = ns, N = 3. Data presented as mean ± SEM. **C** Kymograph of mitochondria within the axonal compartment suggesting increased mitochondrial mobility of SNPH ^−/−^ MNs. **D** Quantification of C. The percentage of moving mitochondria was significantly increased in SNPH ^−/−^ axons. Week1: mean WT = 22%, mean SNPH ^−/−^ = 50.2%. Week3: mean WT = 15.7%, mean SNPH ^−/−^ = 48.5%. Each datapoint represents one axon. Mann–Whitney test, *p* < 0.0001, *N* = 6, *n* = 71–84. Data presented as mean ± SEM. **E** VDAC1 protein levels were not altered between WT and SNPH ^−/−^ three-week-old motor neurons. **F** Quantification of E. Mean WT = 1.00, mean SNPH ^−/−^=1.002, unpaired, parametric t-test, p = ns, N = 3. Data normalised to WT and presented as mean ± SEM

Having established a robust model to study human in-vitro NMJs, we next aimed to determine if axonal mitochondrial transport can modulate NMJ formation and maintenance. Mitochondria, as labelled by VDAC1, were localised at the NMJs of the NMAss (Fig. 2A). To test whether increasing axonal transport of mitochondria would enhance NMJ innervation we next generated a homozygous genetic knockout (KO) of syntaphilin (SNPH), a mitochondrial anchor protein, in hPSCs using CRISPR/Cas9 (Supplementary Fig. 5). Briefly, exon 4 and 5 of *SNPH* were targeted by two gRNAs leading to a 700 bp deletion and, consequently, to an in-frame STOP codon in exon 6 (Supplementary Fig. 5A, B). SNPH KO hPSC line was karyotypically normal (Supplementary Fig. 6) and expressed pluripotency marker (Supplementary Fig. 7). *SNPH* mRNA transcript levels in 3-week-old SNPH^−/−^ MNs were not altered (Supplementary Fig. 5 C), while SNPH protein expression was absent (Supplementary Fig. 5D). Importantly, loss of SNPH did not affect MN differentiation, as assessed by percentage of ISL1/2 positive cells (Fig. 2B, C). To confirm that SNPH loss leads to increased mitochondrial mobility in human stem cell derived MNs we assessed mitochondrial axonal transport by live imaging of MNs transduced with a lentivirus expressing dsRed2 fluorescent protein with mitochondria localisation signal (Supplementary Video 7–8). Mitochondria were considered as motile when their speed was greater than 0.1 µm/s, hence indicating fast axonal transport. As expected, the percentage of moving mitochondria in SNPH^−/−^ MNs was significantly increased (Supplementary Video 7–8, Fig. 2D), while the total amount of VDAC1 protein, a surrogate for total mitochondrial protein, was not altered (Fig. 2E, F).

The speed of motile mitochondria was not markedly different at one week and unaltered at three weeks of culture (Supplementary Fig. 8). In summary, depletion of SNPH increases axonal mitochondrial transport in human MNs.

### Loss of SNPH in SCO increased axonal density in NMAss

**Fig. 3:**
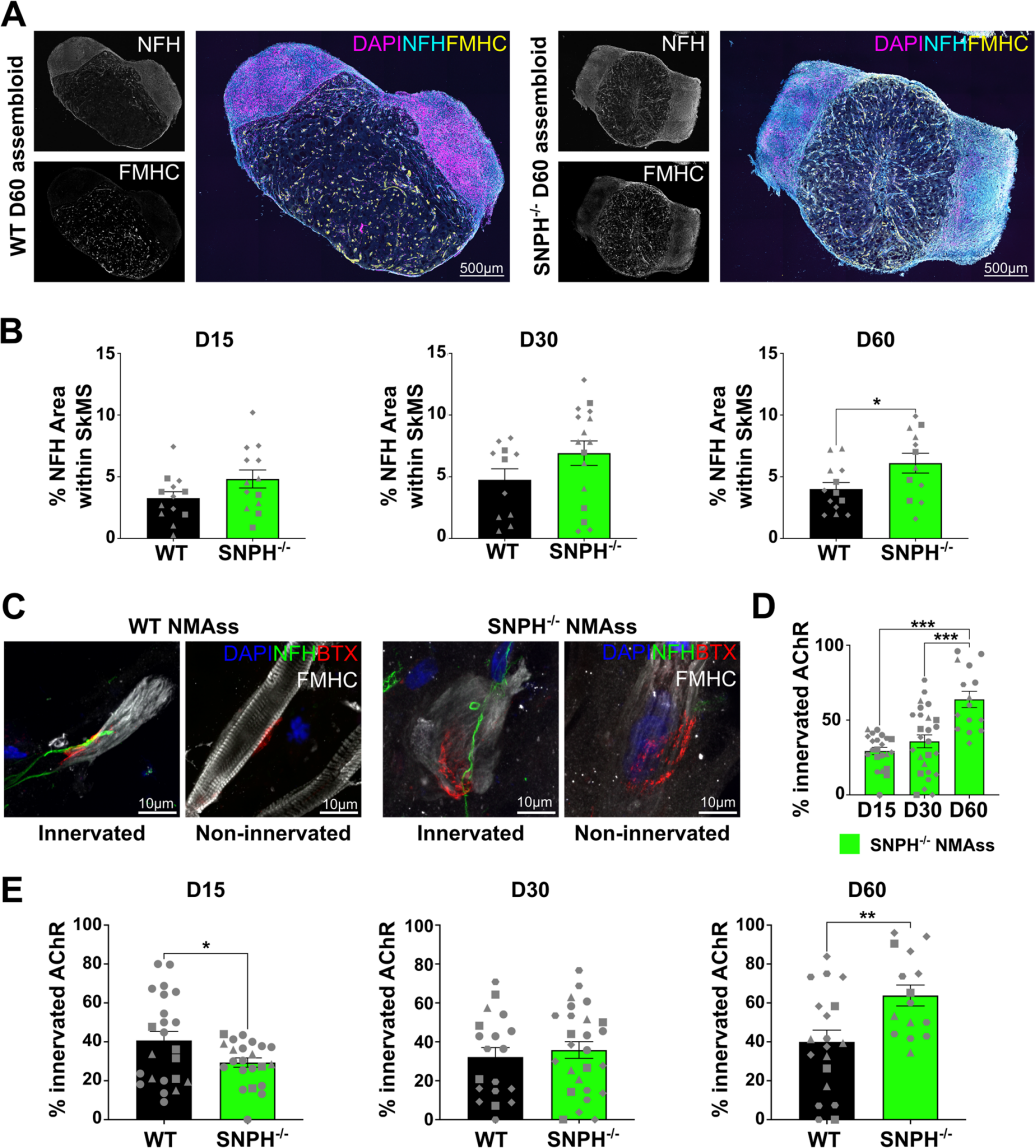
SNPH loss increased axonal density within SkMS region and NMJ innervation. **A** Example image of neuromuscular assembloid. DAPI: nuclei, NFH: axons, FMHC: muscle fibres. **B** The percentage of NFH positive area within SkMS was significantly increased in D60 SNPH ^−/−^ NMAss. Unpaired, parametric t-test, D15 mean WT = 3.3%, mean SNPH ^−/−^ = 4.8%, D30 mean WT = 4.7%, mean SNPH ^−/−^ = 6.9%, D60 mean WT = 4%, mean SNPH ^−/−^ = 6.1%, *p* = ns (D15, D30), *p* = 0.0364 (D60), *N* = 3, *n* = 11–16. Each datapoint represents one NMAss. Different shapes represent different N. **C** Example images of NFH ^+^/BTX ^+^ in-vitro NMJs, and NFH ^−^/BTX ^+^ AChR cluster. Scale bars: 10 µm. NFH: neurofilament heavy chain, BTX: bungarotoxin, FMHC: fast myosin heavy chain. **D** The percentage of NMJ formation in SNPH ^−/−^ NMAss increased over time. Brown Forsythe and Welch’s one-way ANOVA, mean D15 = 29.4%, mean D30: 35.8%, mean D60: 63.8%, *p* = ns (D15, D30), *p* < 0.0001 (D15, D60), *p* = 0.0009 (D30, D60), N = 4–5, n = 15–26. Each datapoint represents one NMAss. Different shapes represent different N. **E** The percentage of innervated AChR cluster was reduced in D15 SNPH ^−/−^ NMAss and increased in D60 SNPH ^−/−^ NMAss. Welch’s t-test, D15: mean WT = 40.7%, mean SNPH ^−/−^ = 29.4%, p = 0.0394; D30: mean WT = 32.2%, mean SNPH ^−/−^ = 35.8%, p = 0.58, D60: mean WT = 40%, mean SNPH ^−/−^ = 63.8%, p = 0.0059, N = 4–5, n = 15–26. Each datapoint represents one NMAss. Different shapes represent different N

Having confirmed that SNPH^−/−^ MNs display increased axonal mitochondrial transport, we next generated SNPH^−/−^ SCOs and NMAss to study the role of SNPH loss on axonal growth, as well as NMJ formation and maintenance. SNPH loss did not alter SCO development, as the number of OLIG2^+^ MN progenitor, ISL1/2^+^ MNs and pan NEUN^+^ neurons (Supplementary Fig. 9A-B) were unchanged. To determine if SNPH loss promotes human MN axonal growth, we next measured axonal density within SNPH^−/−^ NMAss. We observed a significant and time-dependent increase in NFH^+^ axonal density in D60 SNPH^−/−^ NMAss (Fig. 3A, B). In contrast, FMHC^+^ area was not altered between WT and SNPH^−/−^ NMAss (Supplemental Fig. 9 C). In summary, SNPH loss and subsequent increase in mitochondrial axonal transport increased axon growth in NMAss.

### SCO specific SNPH LoF promotes NMJ formation

Next, we studied the effect of SNPH loss on NMJ formation. Temporal assessment of SNPH^−/−^ NMAss, showed a consistent temporal increase in NMJs from D15 to D60 (Fig. 3C, D), which is in contrast to WT (Fig. 1F) where NMJ formation plateaued at D15. We next compared the formation of in-vitro NMJs in WT and SNPH NMAss at each time point. Initially, D15 SNPH^−/−^ AChRs were significantly less innervated than WT (Fig. 3C, E), followed by comparable innervation at D30 and significantly increased innervation at D60 (Fig. 3C, E). Here, selective SNPH LoF within the SCO has shown to promote mitochondrial axonal transport and revealed a so far unreported role in the formation of NMJs.

We next studied if SNPH loss impacts post-synaptic AChR cluster morphology. With the exception of D15 NFH negative ACHR cluster, the post-synaptic size of AChR cluster was unaltered between WT and SNPH (Supplementary Fig. 9D). Similarly, AChR cluster eccentricity, where 0 represents a spherical and 1 a linear shape, was unaltered (Supplementary Fig. 9E). This suggests, that while NMJ formation increased in SNPH^−/−^ NMAss, the AChR morphology was unaltered. In summary, SNPH loss, and therefore increased mitochondrial mobility, promotes MN axonal density in NMAss and, importantly, increases the proportion of NMJs, together suggesting that mitochondrial transport modulates NMJ formation.

## Discussion

In this study we have undertaken a detailed characterisation of a human 3D in-vitro NMJ model and shown that this platform is tractable and can be used to address biological questions. Specifically, we have shown that genetically depleting SNPH results in enhanced NMJ formation.

### The neuromuscular assembloid as a valuable model for human NMJ studies

We described a functional human stem cell derived neuromuscular assembloid model (adapted from Andersen et al. 2020), characterized by ISL1/2^+^ MNs which innervate FMHC^+^ muscle fibres. Human NMJ models are vital for improved mechanistic understanding of human neuromuscular disorders, a point underscored by the recognition of NMJ interspecies differences (Jones et al. 2017; Slater 2017) as well as human specific function of disease-causing genes (Klim et al. 2019; Agra Almeida Quadros et al. 2024; Melamed et al. 2019).

Multiple approaches such as 2D co-cultures, self-organising neuromuscular organoids and assembloids (used in this study) have been adopted in the field (Pereira et al. 2021; Stoklund Dittlau et al. 2021; Massih et al. 2023; Santhanam et al. 2018; Vila et al. 2021; Faustino Martins et al. 2020; Urzi et al. 2023; Andersen et al. 2020; Guo et al. 2020). 2D co-cultures combined with microfluidic technology provides precise control over spatial and temporal organisation of different cell types, enabling functional assessment of NMJs through optogenetics or electrical stimulation of motor neurons (Vila et al. 2021; Guo et al. 2020). 3D co-culture and organoid models facilitate long-term cultures of up to several months due to increased skeletal muscle and AChR maturation (Afshar Bakooshli et al. 2019). 3D self-organising neuromuscular organoids, derived from bi-potent neuromesodermal progenitor cells, provide a unique opportunity to generate neuromuscular organoid models that closely resemble developmental process, as this model self-organises into neuronal and muscle compartments to form NMJs in the presence of Schwann cells (Faustino Martins et al. 2020; Urzi et al. 2023). An advantage of the NMAss model, compared to self-organising neuromuscular organoids, is the ability to study NMJs, composed of muscle and neurons with distinct genetic backgrounds, to address questions of cellular autonomy.

Using the NMAss model, we observed that approximately 40% of AChR cluster were innervated across all the analysed timepoints. Notably, the post-synaptic size of innervated AChR cluster was significantly larger than the post-synaptic size of non-innervated AChR cluster. This observation aligns with findings by Afshar Bakooshli and colleagues who equally used human primary myoblasts in their in-vitro NMJ model (Afshar Bakooshli et al. 2019). In contrast, Couturier and colleagues, employing human stem cell derived myoblasts, reported a reduced AChR size upon MN co-culture (Couturier et al. 2024). These contrasting results may be reflected by the myoblast origin and requires further studies.

We further assessed AChR subunit composition, which are composed of five subunits: two alpha, one beta, one delta and one gamma subunit in fetal AChR (α_2_βδγ), which is replaced by an epsilon subunit in adult AChR (α_2_βδε) (Cetin et al. 2020). In the NMAss model, mRNA analysis showed high expression levels of the alpha-subunit (*CHRNA1)*, the gamma subunit (*CHRNG*) and *MUSK*. Expression of the adult epsilon subunit (*CHRNE)* was less abundant, suggesting that our in-vitro AChRs predominantly contain the fetal γ-AChR subunit. Previous studies showed an upregulation of ε-AChR protein in 3D muscle-neuron co-cultures in comparison to muscle alone, while γ-AChR remained similar (Afshar Bakooshli et al. 2019). This may suggest that the presence of MNs increases adult ε-AChR subunit expression but does not alter the embryonic γ-AChR. Importantly, and in contrast to mice, humans express both adult and fetal AChRs throughout adulthood (Cetin et al. 2020). Future studies are needed to determine the AChR composition within in-vitro NMJs on both RNA and protein level. Taken together, this assembloid model recapitulates key architectural and structural features of the human NMJ*.*

### SNPH loss promotes increased mitochondrial mobility and NMJ formation

Whilst dysfunctional mitochondrial bioenergetics and transport is implicated in neurodegenerative conditions including ALS, the influence of mitochondrial transport and its dysregulation on the NMJ is less well understood.

To enhance bi-directional axonal transport of mitochondria, we genetically depleted SNPH which is an axonal localised protein and functions as a docking protein thereby selectively immobilising mitochondria in the axons (Kang et al. 2008). Consistent with previous findings we observed increased axonal transport of mitochondria in SNPH depleted motor neurons, representing, to our knowledge, the first functional validation of SNPH loss in human motor neurons and, importantly, recapitulating previous findings from rodent studies (Han et al. 2020; Kang et al. 2008; Zhu and Sheng 2011).

Having established a robust model of enhanced mitochondrial transport, we next used the NMAss model to address the impact of SNPH loss on NMJ formation. SNPH was depleted at the stem cell stage, resulting in a global knockout within all cell types of the SCOs. Although SNPH is predominantly expressed in neurons, it has also been found in glial and tumour cells (Kang et al. 2008; Lao et al. 2000; Fu et al. 2023; Nakamura et al. 2023). While it is known that motor neurons selectively innervate skeletal muscle and it is likely that the observed phenotype results from the genetic manipulation within MNs, we cannot exclude a secondary effect of other cell types, which represents a limitation of this study. SCO specific SNPH depletion increased the axonal density within the SkM region of the NMAss. This finding is congruent with other studies supporting that mitochondrial motility is critical to maintain axonal homeostasis in neurons and further promotes axon recovery after injury (Mehta et al. 2021; Han et al. 2020; Zhou et al. 2016). Notably, the impact of SNPH loss extended beyond increased axonal density and resulted in enhanced NMJ formation in SNPH^−/−^ NMAss. The observed increase in AChR innervation may arise by either promoting NMJ maintenance through elevated levels of local ATP and thus providing a higher pool of available energy. Alternatively, SNPH loss may promote terminal axonal branching, suggested by higher axonal density, and is thus able to form more innervated AChRs. Future studies should aim to dissect these mechanisms.

Whilst this study addressed the effects of SNPH loss on NMJ maintenance under physiological conditions, the effect of SNPH loss on NMJ maintenance in a disease context remains to be studied. Axonal transport deficits, particularly of mitochondria, represent a key pathomechanism of ALS (Luan et al. 2024; Saez-Atienzar et al. 2024). Furthermore, NMJ degeneration is one of the earliest pathologies in the disease trajectory of ALS (Verma et al. 2022; Dadon-Nachum et al. 2011; Boido and Vercelli 2016; Fulceri et al. 2021). Therefore, modulating axonal transport might present a potential therapeutic strategy to arrest NMJ degeneration. Further detailed in-vivo and in-vitro studies are required to determine whether SNPH depletion, or alternative approaches promoting enhanced axonal mitochondrial transport, could prevent NMJ degeneration or promote compensatory axonal sprouting in neuromuscular disease such as ALS. These studies will provide a proof-of-concept for identifying therapeutic targets that enhance mitochondrial axonal transport for neuromuscular diseases.

## Conclusions

In summary, we presented an in-depth characterization of a human 3D neuromuscular assembloid model, useful for addressing key biological questions of human NMJ development and disease. NMAss expressed crucial genes of the AChR clustering pathway (Sanes and Lichtman 1999; Burden et al. 2018; Cetin et al. 2020), showed functional innervation and the possibility to long term culture up to 60 days post assembly. Taken together, these findings underscore the relevance of NMAss for future neuromuscular studies. Furthermore, by selective SNPH depletion from SCO, hence increasing axonal mitochondrial transport, we demonstrated a potential role of mitochondrial transport for NMJ maintenance. Future studies might further explore the mechanism underlying increased NMJ innervation, as well as its benefits in a disease context. Ultimately, this study has provided a foundation for future research investigating therapeutic strategies utilising mitochondrial transport to modulate NMJ maintenance.

## Methods

### Human stem cell culture

Human embryonic stem cells (RC17 WT, RC17 SNPH^−/−^, female) were cultured using iPS-Brew culture media (Miltenyi, 130–104-368). The parental human embryonic stem cell line RC17 WT was obtained from the UK Stem cell Bank. Cells were cultured under physiological conditions (37 °C, 5% CO_2_) and medium was exchanged daily. Cells were passaged upon reaching 80–90% confluency, approximately once per week, using 0.5 mM EDTA. Stem cells were regularly confirmed for absence of mycoplasma and for normal karyotype.

### Neuromuscular assembloids

#### Spinal cord organoid differentiation and MN dissociation

Motor neuron (MN) differentiation was performed as per previously published protocol (Mehta et al. 2022; Selvaraj et al. 2018).

At D16, MN spheroids were either dissociated, or cultured for an additional week in D16 + Medium (Advanced DMEM F12 1:2 (Gibco, 12634–010), Neurobasal 1:2 (Gibco, 21103–049), 1 × Anti-anti (Gibco, 15240–062), 1 × Glutamax (Gibco, 35050–038), 1 × N2 (Gibco, 17502–048), 1 × B27 (Gibco, 17504044), 10 µM ascorbic acid (Sigma, A4403), 100 µM beta mercaptoethanol (Gibco, 31350–010) + 100 nM RA (Sigma, R2625), 10 ng/ml BDNF (R&D, 248-BDB), 10 ng/ml GDNF (R&D, 212-GD)) for NMAss generation. Dissociated MNs were seeded according to plate size (coverslips: 26 000 cells/cm^2^, ibidi-dish (µ-Slide 8 Well^High^ ibiTreat, 80806): 50 000 cells/cm^2^, 12-well plate: 214 000 cells/cm^2^) and were fed three times per week with half medium exchange of MN-NF with addition of 1 µM U/FDU for the first week only.

#### Skeletal muscle spheroid generation

Human primary myoblasts (Gibco, A12555, A11440) were seeded in cell culture treated 10 cm dishes at a density of 4 000 cells/cm^2^ in SkM Growth Medium (Promocell, C23060) with medium exchanges every 2–3 days. Upon reaching 80–90% confluency, myoblasts were passaged using 0.05% Trypsin–EDTA (Gibco, 25,200–056). Myoblasts were washed with 1 × PBS and incubated at 37 °C, 5-10 min, with 5 ml of 0.05% Trypsin–EDTA to lift cells off the cell culture dish. To stop the enzymatic reaction, 5 ml of DMEM (Gibco, 41,966–029) was added and cells were transferred to a 15 ml falcon. Cells were centrifuged for 5 min at 400 g, and the cell pellet was resuspended in SkM Growth for further 2D myoblast culture or Geltrex for spheroid generation. For the latter, myoblasts were seeded as SkM spheroids in 20 µl Geltrex (Thermo Fisher, A1413202) at a density of 3 750 cells/µl in ultra-low attachment 96-well round bottom plates (Corning, 7007). Spheroids were allowed to gel at 37 °C for 30 min. Subsequently, 200 µl SkM Growth Medium per well was added and spheroids were cultured on a shaker at 72 rpm (19 mm orbit, ≙0.0628 g). Half Medium changes were performed every 2–3 days. 10 days after lifting, medium was exchanged completely with SkM Diff Medium (Promocell, C23061) with subsequent half medium changes.

#### Neuromuscular assembloids

Spinal cord organoids and SkM spheroids were generated individually and were manually fused on cell culture inserts (Millicell, PICM0RG50) at day 23 (± 2 days) and day 23 (± 7 days), respectively. Assembloids were cultured in neuromuscular assembloid medium (Advanced DMEM F12 (Gibco, 12,634–010), 1 × NEAA (Gibco, 11,140–035), 1 × ITS (Gibco, 51,500–056), 1 × Anti-anti (Gibco, 15,240–062), 10 ng/ml BDNF (R&D, 248-BDB), 10 ng/ml GDNF (R&D, 212-GD), 50 nM cAMP (Sigma, D0627), 200 nM ascorbic acid (Sigma, A4403)) with 3 × medium changes per week.

#### Immunofluorescent staining

2D cell cultures, spinal cord organoids, skeletal muscle spheroids or assembloids were fixed in 4% PFA for 10 min, 30 min, 3 h, 3 h, respectively, at RT and subsequently washed 3 × with 1 × PBS. 3D cultures were cryoprotected by incubation with 30% sucrose in 1 × PBS overnight at 4 °C. Spinal cord organoids were cut into 10 µm thick cryosections, while skeletal muscle spheroids and assembloids were cut into 20 µm cryosections.

Cells were permeabilized with 0.25% Triton X-100 for 10 min, RT, and incubated in block (5% animal serum, 0.25% Triton X-100, PBS) for 1 h, RT. Primary antibodies were applied overnight at 4 °C in blocking solution. Secondary antibodies were applied for 1 h, RT, in blocking solution with DAPI (D9542, Sigma) 1:2000.

For NMJ staining only, auto fluorescent quencher TrueView (Vectorshield, SP-8400) was applied for 5 min, RT.

#### Image acquisition and analysis

Fluorescent images were acquired with a Zeiss LSM 710 confocal microscope using 20x, for SCO and whole NMAss images, or 63x, for NMJ images, objectives. Images were acquired as Z-stacks covering the full Z-depth of a field of view with 1-2 μm distance between focal planes. Z-stacks were compressed into maximum intensity projections and analysed using a combination of Fiji macros and the automated image analysis platform CellProfiler.

#### NMJ analysis

Z-stacks of images containing BTX^+^ AChR cluster were compressed into maximum intensity projections and analysed using Cellprofiler. The thresholding and object detection settings were optimized for each individual channel (NFH, BTX, FMHC) and kept constant for the analysis of an individual batch. NFH^+^, FMHC^+^ and BTX^+^ objects were identified using the IdentifyObject module. In addition, BTX^+^ objects which did not co-localize with FMHC were considered false positive and discarded from the analysis. Each BTX positive object detected by the image analysis pipeline was considered a separate AChR cluster.

Next, BTX^+^ objects were subclassified into innervated and non-innervated objects. Owing to stringent threshold settings for both NFH and BTX objects to avoid false positives, BTX^+^ objects were considered to be innervated if co-localized with or in proximity of 1 µm of NFH objects.

AChR innervation percentage was calculated as number of innervated AChR divided by number of innervated and non-innervated AChR.

#### SV2A/NFH co-localization analysis

Z-Stacks were taken with a 63 × objective on Zeiss LSM 710 and compressed into a maximum intensity projection. BTX^+^ AChR cluster were manually characterised into four subcategories: BTX/NFH/SV2, BTX/NFH, BTX/SV2, BTX. We then calculated the percentage of each subcategory, as well as the percentage of BTX/NFH AChR which were additionally positive for SV2A.

#### Mitochondrial live imaging

Mitochondrial live imaging of 2D MN cultures was performed as previously published in Mehta et al. 2022.

In short, at time point of MN dissociation or 2 weeks prior to imaging, MNs were transduced with a mito-dsRed2 lentivirus at an MOI of 0.1 and seeded at 50 000 cells/cm^2^ in μ-slide 8-well ibidi dishes (Ibidi, 80,826).

Per condition, 12 axons of 2–4 wells were imaged. Videos were aquired using the Zeiss Observer Z1 fluorescent microscope with environmental chamber (5% CO_2_, 37 °C) for 5 min at a frame rate of 3 s and analysed in Fiji using KymoToolBox (https://github.com/fabricecordelieres/IJ_KymoToolBox) with a macro (Supplementary material 1) developed by Laura Murphy (laura.murphy@ed.ac.uk). In short, time laps videos were converted into a 2D plot showing time versus distance of an individual object, here mitochondria. Each objects trajectory was manually traced. The speed of each object was then calculated by dividing the travelled distance by time. Mitochondria moving slower than 0.1 µm/s were labelled as stationary mitochondria.

#### Gene editing

800 000 cells of parental embryonic stem cell line, RC17 WT, passage 30, were nucleofected (Lonza, PBP3-00675, 4D Nucleofector Lonza) with two guide RNAs (2 µg gRNA4, 2 µg gRNA6, Table 1), as well as a 1 µg Puromycin-GFP selection plasmid, and were seeded in 2 wells of a 6-well plate coated with 2.5 µg/ml Laminin 521 (Roche, 11243217001, diluted in PBS containing MgCl_2_ and CaCl_2_ (Sigma, D8662)) in iPSBrew (Miltenyi, 130–104-368) with 10 µM RI (Abcam, Ab120129). Puromycin (1 µg/ml, Sigma, P8833) selection was started after 24 h. Single colonies were picked and screened for a size shift from 1566 to 809 bp. Successful clones were sent for sequencing and karyotyping to exclude the presence of off-target effects. In total, 2% of clones were homozygous for SNPH^−/−^, whereas 12% were heterozygous. For this study, SNPH^−/−^ clone 5 A was used.

**Table 1:** Guide RNA sequence

	Sequence (5’ – 3’)
gRNA4	TAGGCATCCCGCACGCTCAC
gRNA6	GGAAGTAGACGGACCTCTGC

#### Genomic DNA extraction and polymerase chain reaction (PCR)

Genomic DNA was extracted using Bradley lysis buffer (10 mM Tris–HCl ph7.5, 10 mM EDTA, 0.5% SDS, 10 mM NaCl) containing Proteinase K (1:20, Qiagen).

PCR was performed using the Q5 High-Fidelity DNA Polymerase (M0491L). Annealing temperature for individual primer (Table 2) was determined by performing temperature gradient optimization.

**Table 2:** Primer sequence

Target	Primer (5’ – 3’)
SNPH Fw	TGGGGCACATTCACTCATCT
SNPH Rev	AGAAGCCACCTGTGATGACA

#### Western blot

Cells were washed with 1 × PBS and lysed in RIPA buffer (150 mM NaCl, 50 mM Tris–HCl, 0.1% SDS, 0.5% sodium deoxycholate, 1% Triton X-100, 2 mM EDTA, 1 × Protease Inhibitor, pH 7.4). Subsequently, cell lysates were incubated on ice for 15 min and sonicated three times for 5 s, 30% amplitude. Cell debris was separated from the soluble protein fraction by centrifugation at 13 000 g, 4 °C, 15 min. The supernatant was collected, and protein concentration was measured using a Pierce BCA Protein assay (ThermoFisher, 23225). 1 × Laemmli buffer (BioRad, 1610747) was added prior to denaturation at 95 °C, 10 min.

Proteins were separated according to their size via SDS-Gel electrophoresis at 80 V with 1 × Tris–Glycine buffer (24.7 mM Tris, 192 mM Glycine, 3.5 mM SDS). Subsequent protein transfer to a nitrocellulose membrane was performed with 1 × transfer buffer (1 × Tris–Glycine, 20% Methanol) for 2 h, 10 V. Unspecific binding was inhibited by blocking with 5% Milk (Cell signalling, 9999S) in PBS for 1 h, RT, 30 rpm. Primary antibodies in blocking solution were applied to the membranes overnight, 4 °C, 30 rpm. The next day, membranes were washed twice with 1 × TBS (20 mM Tris, 158 mM NaCl, pH 7.6) and once with 1 × TBST (1 × TBS, 0.1% Tween 20) for 15 min, RT, 30 rpm. Appropriate horseradish peroxidase (HRP) conjugated secondaries were applied to the membranes for 1 h, RT, 30 rpm, followed by washing steps as mentioned previously. Enhanced chemiluminescence (ECL) reaction was performed by addition of ECL solution (Cytiva, RPN2105, RPN2235) to the membranes containing HRP conjugated secondaries and developed with an X-ray developer. Images were quantified using Fiji.

### Gene expression analysis

#### RNA extraction and cDNA synthesis

RNA was extracted using the RNeasy Mini Kit (Qiagen, 74,106), according to the manufacturer's instructions. 250 ng of RNA were converted to cDNA using RevertAid (EP0441) reverse transcriptase.

#### qPCR

Gene expression was assessed by quantitative PCR using the DyNAmo Color flash kit (ThermoFisher, F-416) and CFX96 C1000 Touch (BioRad). The annealing temperature for individual primer pairs (Table 3) was determined by performing temperature gradient optimization.

**Table 3:** qPCR primer sequence

Target	Primer (5’ – 3’)	Annealing temperature
18S Fw	GTAACCCGTTGAACCCCATT	61 °C
18S Rev	CCATCCAATCGGTAGTAGCG	
SNPH Exon 5 Fw	CTCAGCAGCAGCAGCAATTC	65.7 °C
SNPH Exon 6 Rev	TGTCACTGCACAGCGTGTAT	

#### RNAscope

RNAscope was performed according to the manufacturer’s instructions using the RNAscope multiplex fluorescent v2 assay (ACD Bio, 323110) with the following alterations. Assembloid fixation was performed for 3 h, 4% PFA, RT, followed by cryoprotection in 30% sucrose overnight. Samples were frozen in 1:1 mix of OCT (Cell Path, KMA-0100-00A) and 30% sucrose solution prior to cryosectioning onto superfrost slides (Epredia, J1800AMNZ) at 20 µm. TSA Vivid dyes were used for visualisation of RNAscope probes and used at a concentration of 1:1500–1:2500. Antibody concentrations were altered from normal immunofluorescent staining and used at the following concentrations. FMHC: 1:100, Goat anti mouse IgG1: 1:400.

Images were taken with a 63 × objective on a Zeiss LSM 710 confocal microscope.

### Antibodies

Tables 4 and 5.

**Table 4:** Primary antibodies

Target	Dilution	Company	Catalogue number	Application
CNX	1:5000	Cell signalling	26,792	WB
FMHC	1:200	Sigma	M1570	IF
GABA	1:500	Novus	NBP2-43,558	IF
ISL1/2	1:100	DSHB	39.4D5c	IF
NFH	1:1000	Biolegend	822,601	IF
NEUN	1:500	Sigma	MAB377	IF
OLIG2	1:1000	Milipore	AB9610,	IF
NANOG	1:250	Cell signaling	3580 s	IF
OCT3/4	1:250	Santa Cruz	sc-5279	IF
MBP	1:100	Abcam	ab7349	IF
PV	1:100	Swant	PV27	IF
S100b	1:100	Abcam	Ab52642	IF
SNPH	1:5000	Abcam	ab192605	WB
SOX9	1:250	Cell signaling	82630S	IF, WB
GFAP	1:500	Abcam	ab4674	IF, WB
VDAC1	1:500, 1:2000	Abcam	ab154856	IF, WB

**Table 5:** Secondary antibodies

Target	Conjugate	Dilution	Company	Catalogue number	Application
Rat	Alexa 488	1:1000	Thermo Fisher	A21208	IF
Rabbit	Alexa 488	1:1000	Thermo Fisher	A11008	IF
Rabbit	HRP	1:5000	Jackson	111–035–144	WB
Chicken	Alexa 488	1:1000	Thermo Fisher	A11039	IF
Chicken	Alexa 555	1:1000	Thermo Fisher	A21437	IF
Mouse IgG1	Alexa 647	1:1000	Thermo Fisher	A21240	IF
Mouse IgG2b	Alexa 555	1:1000	Thermo Fisher	A21147	IF
Bungarotoxin	Alexa 555	1:1000	Thermo Fisher	B35451	IF
DAPI	-	1:2000	Sigma	D9542	IF

#### Statistical analysis

Statistical analysis was performed in Graphpad (Version 10.2.1). For sample size of < 50, normality was assessed using Saphiro-Wilk test. For sample size > 50, normality was assessed using Kolmorgorov-Smirnov. Unless otherwise indicated, equal variance or equal sample size was not assumed, and statistical tests were chosen accordingly. Datasets were analysed as indicated in the figure legend with one of the following statistical tests: parametric t-test (Welch), non-parametric t-test (Mann Whitney), parametric one-way ANOVA (Brown-Forsythe and Welch ANOVA), non-parametric one-way ANOVA (Kruskal–Wallis). Appropriate post-hoc multiple comparison tests were chosen according to the statistical test performed: Dunnet’s T3 (Brown-Forsythe and Welch ANOVA), Dunn (Kruskal–Wallis). Unless otherwise indicated, data is represented as mean ± standard error of mean (SEM). Significance is reached with p < 0.05 (ns = not significant, * = 0.05–0.01, ** = 0.01–0.001, *** < 0.001). N = number of batches, individual conversions; n = number of individual assembloids/organoids. Unless otherwise indicated, different shapes represent different N.

## Supplementary Information

Supplementary Material 1.

Supplementary Video 1. Muscle contraction in WT NMAss.

Supplementary Video 2. Spontaneous calcium flux in WT NMAss.

Supplementary Video 3. Spontaneous calcium flux in WT NMAss.

Supplementary Video 4. Spontaneous calcium flux in WT NMAss.

Supplementary Video 5. Spontaneous muscle contraction in WT NMAss is stopped by Curare treatment.

Supplementary Video 6. D30 Skeletal muscle organoids do not exhibit spontaneous muscle contractions.

Supplementary Video 7. Mitochondrial imaging in WT MNs.

Supplementary Video 8. Mitochondrial imaging in SNPH KO MNs.

Supplemental Figure 1. Characterization of SCO and SkMS. A: Immunocytochemistry of D9 SCO showed enrichment of motor neuron progenitor OLIG2. Scale bar: 100µm. B: D24 SCO was highly enriched in ISL1/2, motor neuron marker, and NEUN, pan-neuronal marker. Scale bar: 200µm. C: D24 SCOs contain a low amount of GABA+ neurons, but no PV+ neurons. Scale bar: 100µm. D: D9 and D24 SCOs were negative for S100B. Scale bar: 100µm. E: D24 and D56 SCO showed few SOX9+ cells and little GFAP+ astrocytic processes in D56 SCO. Scale bar: 100µm. F: Immunocytochemistry of MBP was negative in D24 and D56 SCO. Scale bar: 100µm. G: D24 SkMS contained FMHC+ and TITIN+ skeletal muscle fibres. Scale bar: 100µm. G’: Expression of muscle marker MYOD, DESMIN and TITIN in D24 SkMS. Scale bar: 25µm.

Supplemental Figure 2. Characterization of cellular composition of NMAss. A: NEUN+ and ISL1/2+ motor neurons within the SCO region of NMAss at D30. Scale bar: 200µm, 500µm. B: In D30 NMAss, SOX9+ and GFAP+ astrocytes were detected. Scale bar: 500µm, 10µm. C: D30 NMAss contained MBP+ oligodendrocytes. Scale bar: 500µm. High magnification inset showing the morphology of MBP+ cell. Scale bar: 10µm. D: Expression of DESMIN and TITIN within SkMS of D30 NMAss. Scale bar: 100µm.

Supplemental Figure 3. In-vitro NMJs co-localize with NFH and SV2A pre-synaptic marker while AChR in SkMS were not innervated. A: Representative image of in-vitro NMJs with pre-synaptic NFH and SV2A, as well as post-synaptic AChR visualised by BTX. Scale bar: 10µm. B: 94.8% of in-vitro NMJs positive for NFH and BTX, are also positive for SV2A. 30.8% of non-innervated AChR (NFH-/BTX+) are positive for SV2A. N=3, n=10. C: Pie chart showing the percentage of NMAss/SkMS containing BTX staining. Age of D+30 SkMS was comparable to age of SkMS within D30 NMAss. All NMAss presented with BTX+ areas, whereas SkMS alone was not consistently positive for BTX. Unpaired, parametric t-test with Welch’s correction, mean WT=100%, mean SkMS= 46.6%, p=ns, N=5, n=24 (NMAss), n=17 (SkMS). D: BTX area of non-innervated AChR cluster was comparable between NMAss and SkMS. Unpaired, parametric t-test with Welch’s correction, mean NMAss=8.2µm2, mean SkMS=7.6 µm2, p=ns, N=2, n=5-10. Each datapoint represents one NMAss/SkMS. Different shapes represent different N. Data presented as mean ± SEM. E: As excpected, SkMS were negative for NFH and hence did not form innervated AChR cluster. Unpaired, parametric t-test with Welch’s correction, mean NMAss=33.3%, mean SkMS=0%, p=0.0006, N=2, n=5-10. Each datapoint represents one NMAss/SkMS. Different shapes represent different N. Data presented as mean ± SEM.

Supplemental Figure 4. Temporal analysis of NMJ specific genes in human NMAss model. A-B: Expression of CHRNA1, CHRNE, CHRNG and MUSK in D30 (A) and D60 (B) NMAss alongside with FMHC protein staining and DAPI nuclear counterstain. Scale bars: 25µm. C: Negative controls ensure specificity of RNAscope probes as no positive signal was observed using an RNAscope probe against bacterial DapB, no secondary or no probe controls. Scale bar: 25µm

Supplemental Figure 5. Generation and validation of SNPH-/- embryonic stem cell line. A: Schematic of CRISPR Cas9 approach. Two cut sites were introduced in exon 4 and exon 5, leading to a truncated exon4/exon5 DNA sequence and to a premature stop codon within the amino acid sequence of exon 6. B: Agarose gel confirming deletion of 700bp in SNPH-/- hPSCs (WT: 1.5kb, SNPH-/-: 800bp). C: Gene expression of SNPH in 3-week-old MN cultures was not altered upon gene editing. Mean WT=1.04, mean SNPH-/-=1.2. Unpaired, parametric t-test, p=ns, N=3. Data normalised to WT and presented as mean ± SEM. D: SNPH-/- leads to a complete loss of SNPH protein in 3-week-old motor neurons. Mean WT=1.0, mean SNPH-/-=0.03. Unpaired, parametric t-test, p=0.0499, N=3. Data normalised to WT and presented as mean ± SEM.

Supplemental Figure 6. Karyotype analysis with array comparative genomic hybridization technique. Results showed normal karyotype of SNPH-/- embryonic stem cell line, clone 5A, in RC17 WT background. 

Supplemental Figure 7. Pluripotency immunocytochemistry of WT (A) and SNPH-/- (B) embryonic stem cell lines. Immunocytochemistry of NANOG and OCT3/4 staining, two pluripotency marker, in embryonic stem cells, at passage 35 and 47 for WT and SNPH-/-, respectively. Scale bar: 100µm.

Supplemental Figure 8. Speed of moving mitochondria. Speed of moving mitochondria (µm/s) was significantly different between WT and SNPH-/- MNs of (A) one-week-old motor neurons, but unaltered at (B) three-weeks. Each datapoint represents one mitochondrion. Mann-Whitney test, one week: mean WT=0.69mm/s, mean SNPH-/-=0.56mm/s, p=0.0002, N=6, n=245-502; three weeks: mean WT=1.13mm/s, mean SNPH-/-=0.95mm/s, p=0.36, N=6, n=232-614.

Supplemental Figure 9. SCO differentiation, SkM area and AChR size were not altered upon SNPH loss. A: Loss of SNPH did not lead to altered numbers of OLIG2+ neuronal progenitor. DAPI: nuclei, OLIG2: motor neuron progenitor. Scale bar: 100 µm. Mann-Whitney test, WT=31.2%, SNPH-/-=32.2%, p=ns, N=3, n=12-13. Each datapoint represents one SCO. Different shapes represent different N. B: Loss of SNPH did not lead to altered numbers of ISL1/2+ or NEUN+ neurons. An eccentricity of 0 represents a spherical shape, while eccentricity of 1 represents a linear shape. DAPI: nuclei, ISL1/2: motor neuron marker, NEUN: pan-neuronal marker. Scale bar: 200µm. Mann-Whitney test, ISL1/2: WT=36.3%, SNPH-/-=38%, NEUN: WT=80.2%, SNPH-/-=82.7%, p=ns, N=4, n=19. Each datapoint represents one SCO. Different shapes represent different N. C: The percentage of FMHC+ area within SkMS of NMAss was not altered. Unpaired, parametric t-test, p=ns (D15, D60), Mann-Whitney test, D15 mean WT=10.6%, mean SNPH-/-=11.3%, D30 mean WT=10.7%, mean SNPH-/-=13.1%, D60 mean WT=7.0%, mean SNPH-/-=7.7%, p=ns (D30), N=2-3, n=8-16. Each datapoint represents one NMAss. Different shapes represent different N. Data presented as mean ± SEM. D: Quantification of post-synaptic BTX+ area of innervated or non-innervated AChR cluster showed no significant difference between genotypes with the exception of D15 non-innervated AChRs. Welch’s t-test or Mann-Whitney test comparing innervated or non-innervated AChR cluster between genotypes, NFH+/BTX+ AChR cluster mean D15: WT= 15.3µm2, SNPH-/-=13.3µm2, p=0.67, mean D30: WT=15.4µm2, SNPH-/-=13.5µm2, p=0.39, mean D60: WT=12.4µm2, SNPH-/-=12µm2, p=0.82, NFH-/BTX+ AChR cluster mean D15: WT=8.5µm2, SNPH-/=10.5µm2, p=0.04, mean D30: WT=7.3µm2, SNPH-/-=8.7µm2, p=0.37, mean D60: WT=7.5µm2, SNPH-/-=9µm2, p=0.89, N=4-5, n=15-26. Each datapoint represents one NMAss. Different shapes represent different N. Data presented as mean ± SEM. E: Eccentricity of BTX+ AChR cluster was not altered between genotypes. An eccentricity of 0 represents a spherical shape, while eccentricity of 1 represents a linear shape. NFH+/BTX+ AChR cluster mean D15: WT=0.88 , SNPH-/-=0.88, p=ns, mean D30: WT=0.89, SNPH-/-=0.87, p=ns, mean D60: WT=0.89, SNPH-/-=0.88, p=ns, NFH-/BTX+ AChR cluster mean D15: WT=0.84, SNPH-/=0.87, p=ns, mean D30: WT=0.88, SNPH-/-=0.86, p=ns, mean D60: WT=0.84, SNPH-/-=0.88, p=ns, N=4-5, n=15-26, Welch’s t-test or Mann-Whitney test. Each datapoint represents one NMAss. Different shapes represent different N. Data presented as mean ± SEM.

## Data Availability

The datasets used and/or analysed during the current study are available from the corresponding author on reasonable request.

## Abbreviations

Ach: Acetylcholine
AChR: Acetylcholine Receptor
ALS: Amyotrophic Lateral Sclerosis
BDNF: Brain-Derived Neurotrophic Factor
Bp: Basepair
BTX: Bungarotoxin
cDNA: Complementary DNA
CNX: Calnexin
D: Day
DD: Degradation Domain
DMEM: Dulbecco's Modified Eagle Medium
DNA: Deoxyribonucleic Acid
ECL: Enhanced Chemiluminescence
EDTA: Ethylenediaminetetraacetic Acid
fALS: Familial ALS
FMHC: Fast Myosin Heavy Chain
FUS: Fused in Sarcoma
GDNF: Glial-Derived Neurotrophic Factor
GFP: Green Fluorescent Protein
GoF: Gain of Function
gRNA: guide RNA
HRP: Horseradish Peroxidase
IF: Immunofluorescent
ISL1/2: Islet1/2
kDa: Kilo Dalton
KO: Knockout
LoF: Loss of Function
MBP: Myelin basic protein
MN: Motor Neuron
MN Diff base: Motor Neuron Differentiation Medium
MN NF: Motor Neuron Neurotrophic Factors
MOI: Multiplicity of Infection
mRNA: messenger RNA
MUSK: Muscle Specific Kinase
NEUN: Neuronal nuclear antigen
NFH: Neurofilament heavy chain
NMAss: Neuromuscular Assembloid
NMJ: Neuromuscular Junction
OLIG2: Oligodendrocyte Transcription Factor 2
PBS: Phosphate-Buffered Saline
PCR: Polymerase Chain Reaction
PFA: Paraformaldehyde
RA: Retinoic Acid
RNA: Ribonucleic Acid
RT: Room Temperature
SCO: Spinal Cord Organoid
SDS: Sodium Dodecyl Sulfate
Sh: Shield
SkM: Skeletal Muscle
SkMS: Skeletal Muscle Spheroid
SNPH: Syntaphilin
STMN2: Stathmin 2
TARDBP: Transactive Response DNA Binding Protein
WT: Wildtype
