# Supplementary material for: Syntaphilin loss enhances mitochondrial axonal transport and neuromuscular junction formation in a human stem cell derived neuromuscular assembloid model
Source: Mol Med. 2025 Nov 5;31:328. doi: 10.1186/s10020-025-01319-x (PMC12590865; doi:10.1186/s10020-025-01319-x)
Supplement: Supplementary file 1 — Supplementary Material 1. [file 10020_2025_1319_MOESM1_ESM.docx]

/*

* Macro written

* Initially, user is asked for folders containing czi folder and a folder where they want results saved

* User traces along axon and KymoToolBox plugin (https://github.com/fabricecordelieres/IJ-Plugin_KymoToolBox) is used to create a kymograph.

* User then traces the tracks on the kymograph which are also analysed using the plugin

* Results are saved in a folder for each image and a summary spreadsheet is saved in the outer results folder

*

* Written by Laura Murphy (laura.murphy@ed.ac.uk)

* IGMM Advanced Imaging Resource

* Nov 2020

*/

//--------------------------------//-----------------------------------------------------------------------------------

//-- Part 0: Preparation steps: get directories from users and setting up arrays

//--------------------------------//-----------------------------------------------------------------------------------

// -- Getting input and output folders from user input as well as parameters for kymotoolbox

inputFolder = getDirectory("Choose the folder containing your images");

outputFolder = getDirectory("Choose the folder where you want to save your results")

title = "KymoToolBox Settings";

width=512; height=512;

Dialog.create("KymoToolBox Settings");

Dialog.addMessage("\nSettings for kymograph");

Dialog.addNumber("Kymograph Width (microns) :", 20);

Dialog.addNumber("Speed Limit (microns/sec) :", 0.1);

Dialog.addMessage("\n Display settings for output");

Dialog.addNumber("Line thickness (pixels) :", 2);

Dialog.addNumber("Dot size (pixels) :", 10);

Dialog.show();

kymoWidth = Dialog.getNumber();

speedLimit = Dialog.getNumber();

lineThickness = Dialog.getNumber();

dotSize = Dialog.getNumber();

// -- Produce list to only be .czi

dirList = newArray();

dirList = getFileTree(inputFolder, dirList);

// -- Message to user about how many images will be processed

count = dirList.length

print("There are " + count + " files to be processed");

// -- Create arrays for saving later

Filename = newArray();

Mitochondria = newArray();

TotalTime_Secs = newArray();

CumulDist_Microns = newArray();

MeanSpeed_MicronPerSec = newArray();

Classification = newArray();

//--------------------------------//-----------------------------------------------------------------------------------

//-- Part 1: Opening images and creating results directories

//--------------------------------//-----------------------------------------------------------------------------------

// -- Using Bio-Formats to open images

for (i = 0; i < dirList.length; i++){

path = dirList[i];

run("Bio-Formats Macro Extensions");

run("Bio-Formats Importer", "open=&path color_mode=Composite rois_import=[ROI manager] view=Hyperstack stack_order=XYCZT series_1");

fileName = getTitle();

imgName = File.nameWithoutExtension();

print("Current file is: " + fileName);

getDimensions(width, height, channels, slices, frames);

if(channels > 1){

run("Split Channels");

selectWindow("C1-" + fileName);

rename(fileName);

selectWindow("C2-" + fileName);

run("Close");

selectWindow("C3-" + fileName);

run("Close");

}

imgFolder = outputFolder + File.separator + imgName + File.separator;

File.makeDirectory(imgFolder);

getDimensions(width, height, channels, slices, frames);

getPixelSize(unit, pixelWidth, pixelHeight);

//--------------------------------//-----------------------------------------------------------------------------------

//-- Part 2: File processing, get user to trace axon and mitochondria

//--------------------------------//-----------------------------------------------------------------------------------

selectWindow(fileName);

run("Grays");

run("Z Project...", "projection=[Max Intensity]");

setTool("polyline");

setMinAndMax(100, 1500);

waitForUser("Trace your axon length that the kymograph will be created from and click 'OK' once done\n\n ");

if (selectionType == -1){

print("No line drawn, image will be skipped");

run("Close All");

continue;

}

roiManager("Add");

roiManager("Save", imgFolder + "Axon.roi");

roiManager("Reset");

run("Measure");

selectWindow(fileName);

run("Duplicate...", "use");

run("RGB Color");

run("Restore Selection");

length = getResult("Length", 0);

setColor("white");

x=1000; y=200;

drawString("Axon length: " + length + " microns", x, y);

run("Flatten", "slice");

saveAs("Tiff", imgFolder + imgName + "_tracedAxon.tiff");

run("Clear Results");

run("Close");

selectWindow("MAX_" + fileName);

run("Close");

selectWindow(fileName);

run("Properties...", "channels=1 slices=" + frames + " frames=" + slices + " pixel_width=" + pixelWidth + " pixel_height=" + pixelHeight + " voxel_depth=1.0000000 frame=[3.00 sec]");

run("Restore Selection");

run("Draw Kymo", "width=" + kymoWidth + " get_kymo get_kymostack get_kymomontage");

selectWindow("Kymograph from " + fileName);

run("Duplicate...", " ");

rename(fileName + "_kymo");

saveAs("Tiff", imgFolder + imgName + "_kymograph.tiff");

run("Close");

selectWindow("KymoStack from " + fileName);

saveAs("Tiff", imgFolder + imgName + "_straightened.tiff");

run("Close");

selectWindow("KymoMontage from " + fileName);

saveAs("Tiff", imgFolder + imgName + "_montage.tiff");

run("Close");

selectWindow("Kymograph from " + fileName);

setMinAndMax(100, 1000);

setTool("polyline");

waitForUser("Trace your mitochondria, clicking 't' each time one is complete. Once they all traced click 'OK'");

//--------------------------------//-----------------------------------------------------------------------------------

//-- Part 3: Saving results and storing for summaries

//--------------------------------//-----------------------------------------------------------------------------------

for (n = 0; n < roiManager("Count"); n++){

roiManager("Select", n);

roiManager("rename", n+1);

Filename = Array.concat(Filename,imgName);

Mitochondria = Array.concat(Mitochondria, n+1);

}

run("Analyse Kymo", "outward=[From left to right] lim.=" + speedLimit + " line=" + lineThickness + " log_all_data log_extrapolated_coordinates show report original=[" + fileName + "] dot=" + dotSize);

wait(2000);

selectWindow("Composite");

run("Flatten");

saveAs("Tiff", imgFolder + imgName + "_colouredDots.tiff");

run("Close");

selectWindow("Tracks from Kymograph from " + fileName);

saveAs("Tiff", imgFolder + imgName + "_annotatedKymograph.tiff");

run("Close");

selectWindow("Results");

saveAs("Results", imgFolder + imgName + "_FullResults.csv");

for (r = 0; r < nResults; r++){

label = getResultLabel(r);

if (matches(label, ".*Summary.*")) {

res1 = getResult("Ttl_Time_(sec)", r);

TotalTime_Secs = Array.concat(TotalTime_Secs, res1);

res2 = getResult("Cum_Dist_(micron)", r);

CumulDist_Microns = Array.concat(CumulDist_Microns, res2);

res3 = getResult("Mean_Speed_(micron_per_sec)", r);

MeanSpeed_MicronPerSec = Array.concat(MeanSpeed_MicronPerSec, res3);

if (res3 < 0.1){

Classification = Array.concat(Classification, "Static");

} else {

Classification = Array.concat(Classification, "Moving");

}

}

}

roiManager("Deselect");

roiManager("save", imgFolder + imgName + "_KymoROIs.zip");

//--------------------------------//-----------------------------------------------------------------------------------

//-- Part 5: Closing loop and finishing off

//--------------------------------//-----------------------------------------------------------------------------------

run("Clear Results");

run("Close All");

roiManager("Reset");

run("Collect Garbage");

Array.show(Filename, Mitochondria, TotalTime_Secs, CumulDist_Microns, MeanSpeed_MicronPerSec, Classification);

saveAs("Results", outputFolder + "Macro Summary Results.csv");

run("Close");

}

// -- Save the average results for each image

print("\\Clear");

Array.show(Filename, Mitochondria, TotalTime_Secs, CumulDist_Microns, MeanSpeed_MicronPerSec, Classification);

saveAs("Results", outputFolder + "Macro Summary Results.csv");

Dialog.create("Progress");

Dialog.addMessage("Macro Complete!");

Dialog.show;

//--------------------------------//-----------------------------------------------------------------------------------

//-- Epilogue: Functions

//--------------------------------//-----------------------------------------------------------------------------------

function getFileTree(dir , fileTree){

list = getFileList(dir);

for(f = 0; f < list.length; f++){

if (matches(list[f], "(?i).*\\.(tiff|tif|czi)$"))

fileTree = Array.concat(fileTree, dir + list[f]);

if(File.isDirectory(dir + File.separator + list[f]))

fileTree = getFileTree(dir + list[f],fileTree);

}

return fileTree;

}
