# Supplementary figures and images for: Syntaphilin loss enhances mitochondrial axonal transport and neuromuscular junction formation in a human stem cell derived neuromuscular assembloid model
Source: Mol Med. 2025 Nov 5;31:328. doi: 10.1186/s10020-025-01319-x (PMC12590865; doi:10.1186/s10020-025-01319-x)

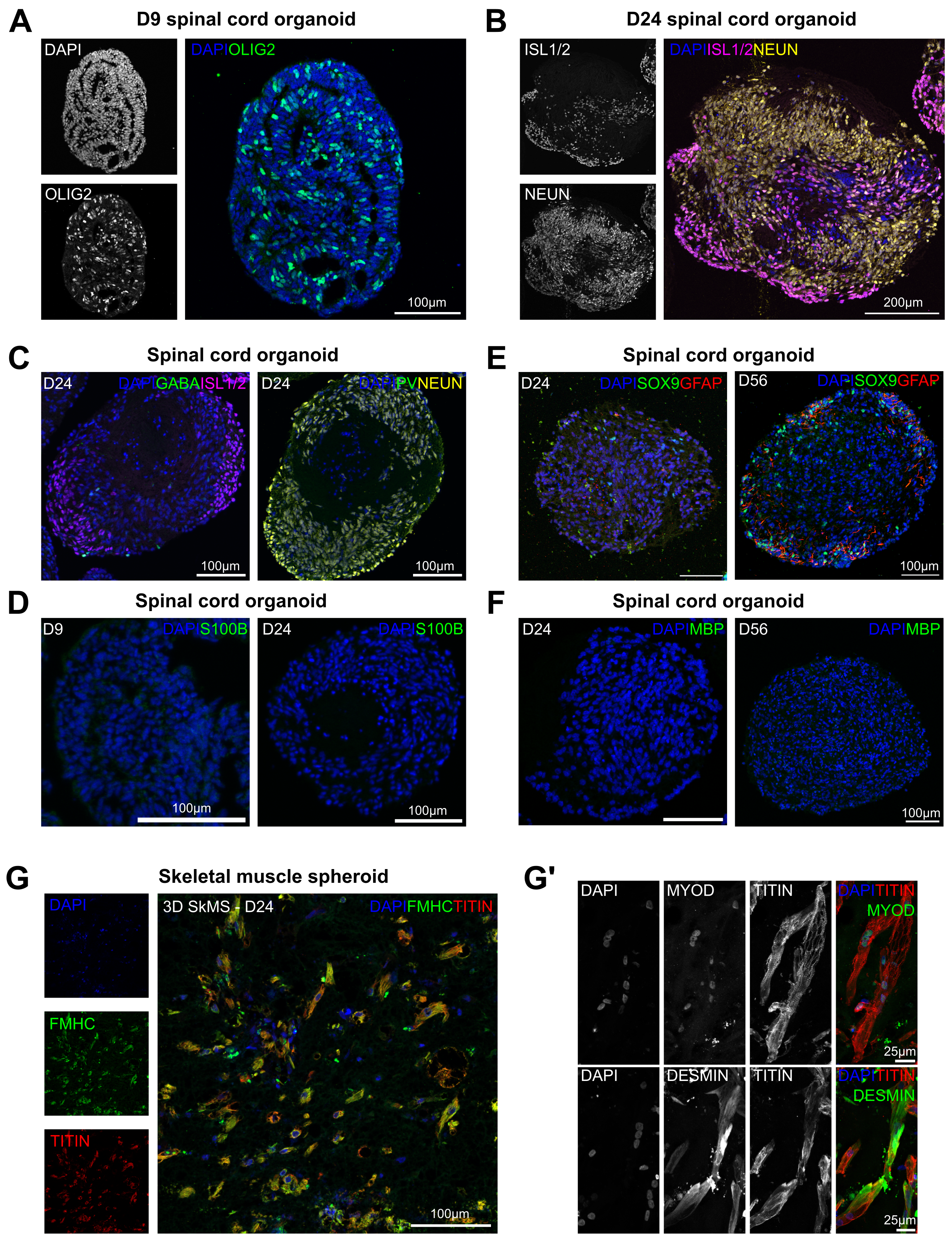

Supplement: Supplementary file 10 — Supplemental Figure 1. Characterization of SCO and SkMS. A: Immunocytochemistry of D9 SCO showed enrichment of motor neuron progenitor OLIG2. Scale bar: 100µm. B: D24 SCO was highly enriched in ISL1/2, motor neuron marker, and NEUN, pan-neuronal marker. Scale bar: 200µm. C: D24 SCOs contain a low amount of GABA+ neurons, but no PV+ neurons. Scale bar: 100µm. D: D9 and D24 SCOs were negative for S100B. Scale bar: 100µm. E: D24 and D56 SCO showed few SOX9+ cells and little GFAP+ astrocytic processes in D56 SCO. Scale bar: 100µm. F: Immunocytochemistry of MBP was negative in D24 and D56 SCO. Scale bar: 100µm. G: D24 SkMS contained FMHC+ and TITIN+ skeletal muscle fibres. Scale bar: 100µm. G’: Expression of muscle marker MYOD, DESMIN and TITIN in D24 SkMS. Scale bar: 25µm. [file 10020_2025_1319_MOESM10_ESM.tiff]

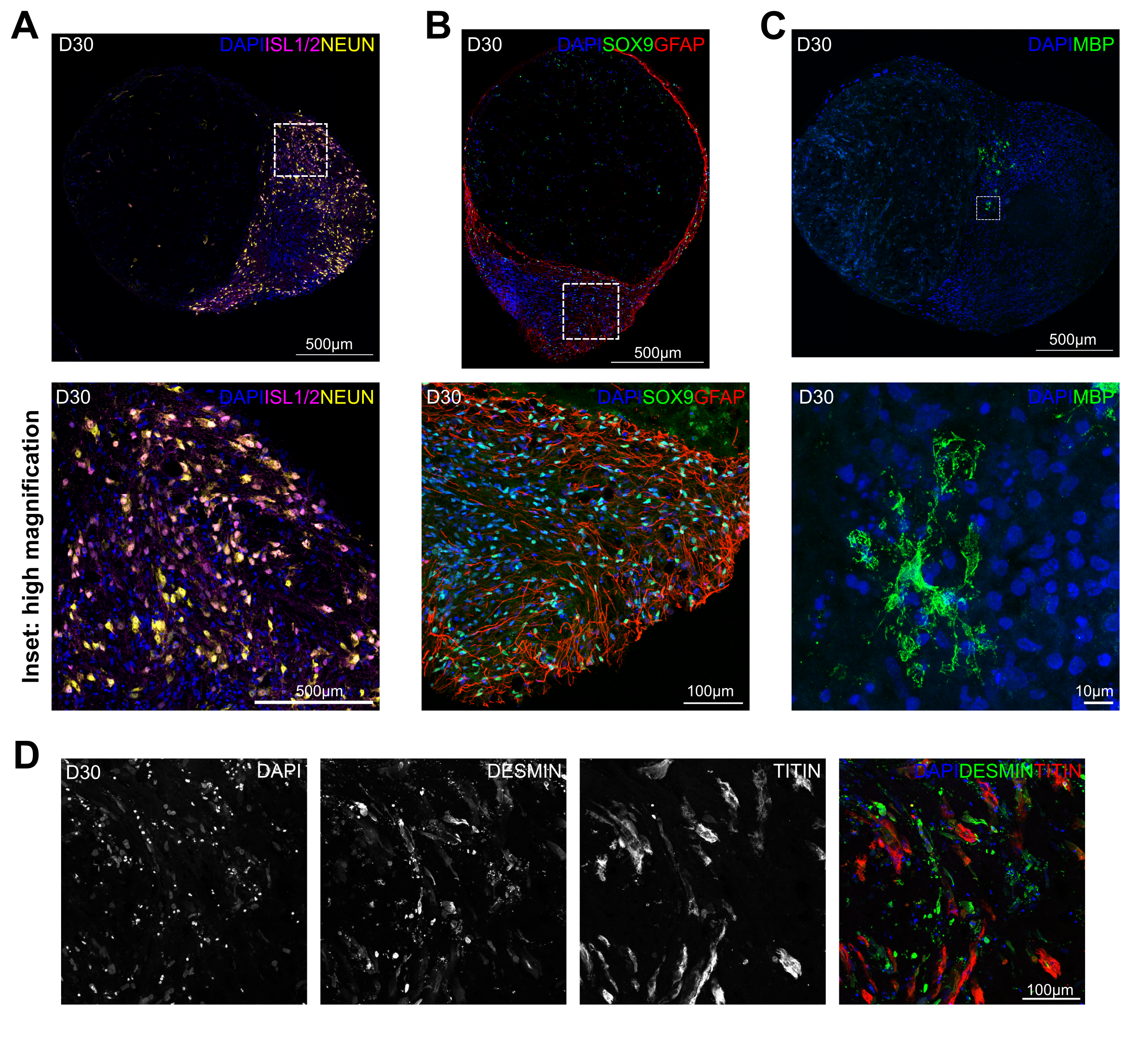

Supplement: Supplementary file 11 — Supplemental Figure 2. Characterization of cellular composition of NMAss. A: NEUN+ and ISL1/2+ motor neurons within the SCO region of NMAss at D30. Scale bar: 200µm, 500µm. B: In D30 NMAss, SOX9+ and GFAP+ astrocytes were detected. Scale bar: 500µm, 10µm. C: D30 NMAss contained MBP+ oligodendrocytes. Scale bar: 500µm. High magnification inset showing the morphology of MBP+ cell. Scale bar: 10µm. D: Expression of DESMIN and TITIN within SkMS of D30 NMAss. Scale bar: 100µm. [file 10020_2025_1319_MOESM11_ESM.tiff]

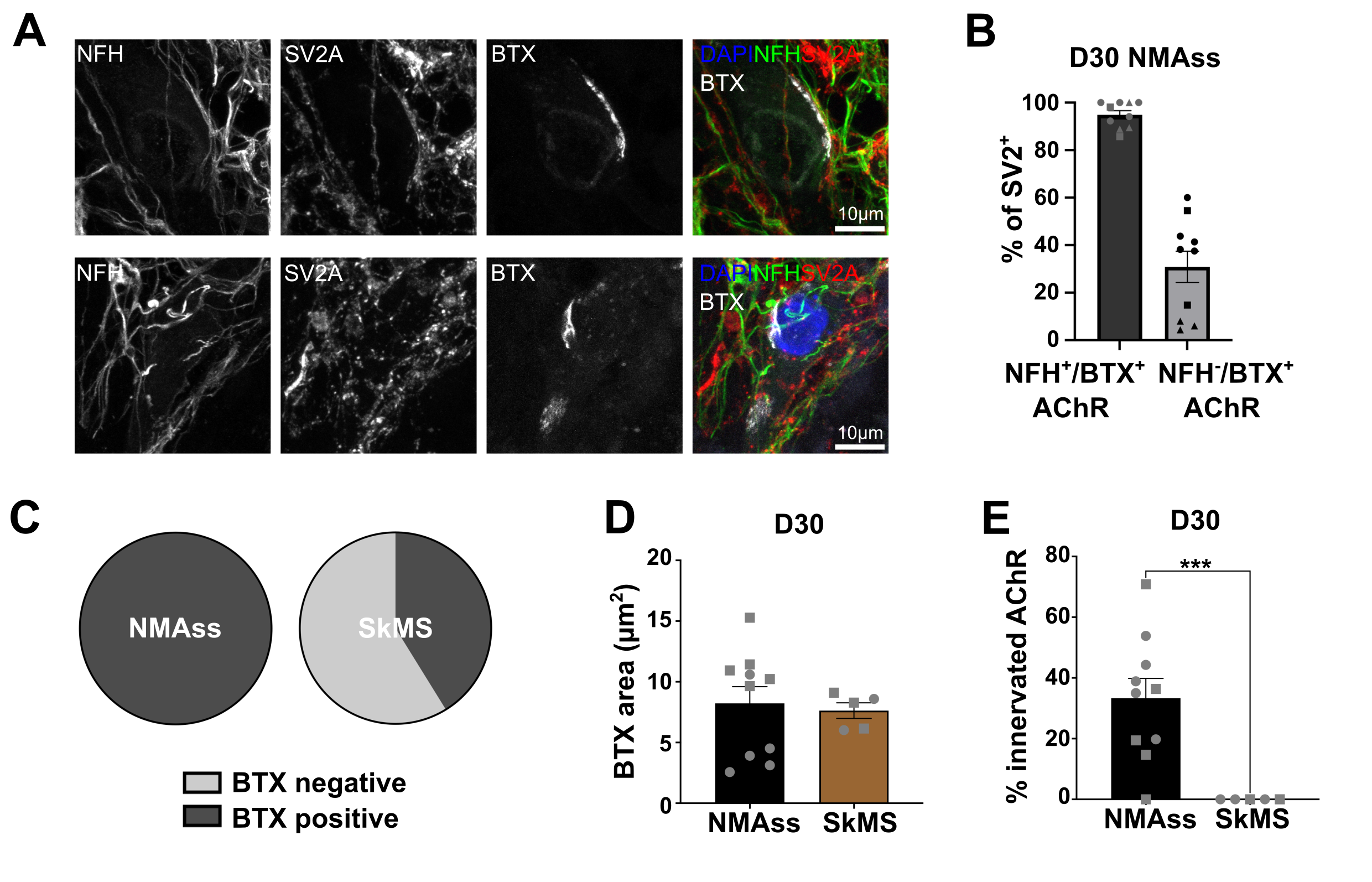

Supplement: Supplementary file 12 — Supplemental Figure 3. In-vitro NMJs co-localize with NFH and SV2A pre-synaptic marker while AChR in SkMS were not innervated. A: Representative image of in-vitro NMJs with pre-synaptic NFH and SV2A, as well as post-synaptic AChR visualised by BTX. Scale bar: 10µm. B: 94.8% of in-vitro NMJs positive for NFH and BTX, are also positive for SV2A. 30.8% of non-innervated AChR (NFH-/BTX+) are positive for SV2A. N=3, n=10. C: Pie chart showing the percentage of NMAss/SkMS containing BTX staining. Age of D+30 SkMS was comparable to age of SkMS within D30 NMAss. All NMAss presented with BTX+ areas, whereas SkMS alone was not consistently positive for BTX. Unpaired, parametric t-test with Welch’s correction, mean WT=100%, mean SkMS= 46.6%, p=ns, N=5, n=24 (NMAss), n=17 (SkMS). D: BTX area of non-innervated AChR cluster was comparable between NMAss and SkMS. Unpaired, parametric t-test with Welch’s correction, mean NMAss=8.2µm2, mean SkMS=7.6 µm2, p=ns, N=2, n=5-10. Each datapoint represents one NMAss/SkMS. Different shapes represent different N. Data presented as mean ± SEM. E: As excpected, SkMS were negative for NFH and hence did not form innervated AChR cluster. Unpaired, parametric t-test with Welch’s correction, mean NMAss=33.3%, mean SkMS=0%, p=0.0006, N=2, n=5-10. Each datapoint represents one NMAss/SkMS. Different shapes represent different N. Data presented as mean ± SEM. [file 10020_2025_1319_MOESM12_ESM.tiff]

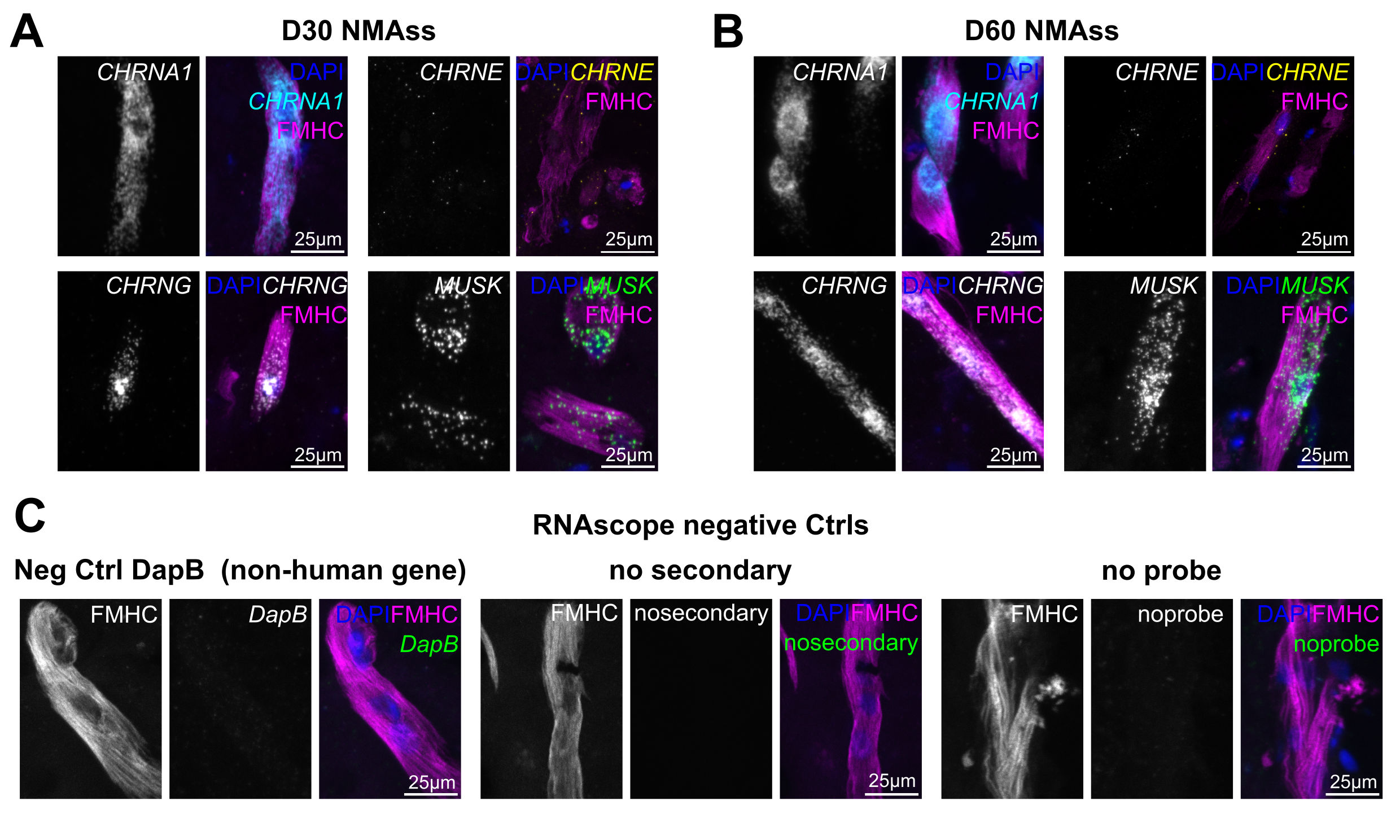

Supplement: Supplementary file 13 — Supplemental Figure 4. Temporal analysis of NMJ specific genes in human NMAss model. A-B: Expression of CHRNA1, CHRNE, CHRNG and MUSK in D30 (A) and D60 (B) NMAss alongside with FMHC protein staining and DAPI nuclear counterstain. Scale bars: 25µm. C: Negative controls ensure specificity of RNAscope probes as no positive signal was observed using an RNAscope probe against bacterial DapB, no secondary or no probe controls. Scale bar: 25µm [file 10020_2025_1319_MOESM13_ESM.tiff]

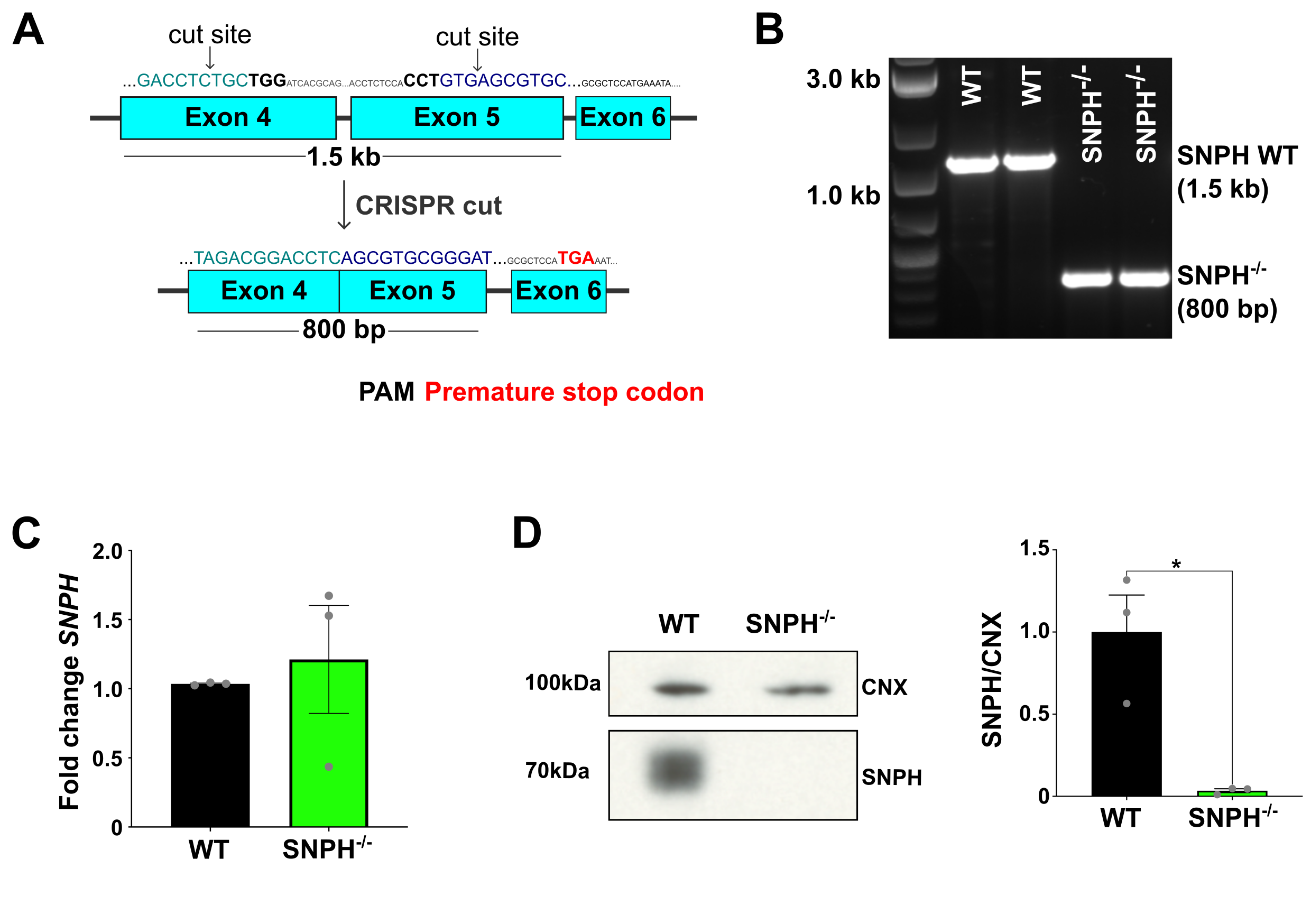

Supplement: Supplementary file 14 — Supplemental Figure 5. Generation and validation of SNPH-/- embryonic stem cell line. A: Schematic of CRISPR Cas9 approach. Two cut sites were introduced in exon 4 and exon 5, leading to a truncated exon4/exon5 DNA sequence and to a premature stop codon within the amino acid sequence of exon 6. B: Agarose gel confirming deletion of 700bp in SNPH-/- hPSCs (WT: 1.5kb, SNPH-/-: 800bp). C: Gene expression of SNPH in 3-week-old MN cultures was not altered upon gene editing. Mean WT=1.04, mean SNPH-/-=1.2. Unpaired, parametric t-test, p=ns, N=3. Data normalised to WT and presented as mean ± SEM. D: SNPH-/- leads to a complete loss of SNPH protein in 3-week-old motor neurons. Mean WT=1.0, mean SNPH-/-=0.03. Unpaired, parametric t-test, p=0.0499, N=3. Data normalised to WT and presented as mean ± SEM. [file 10020_2025_1319_MOESM14_ESM.tiff]

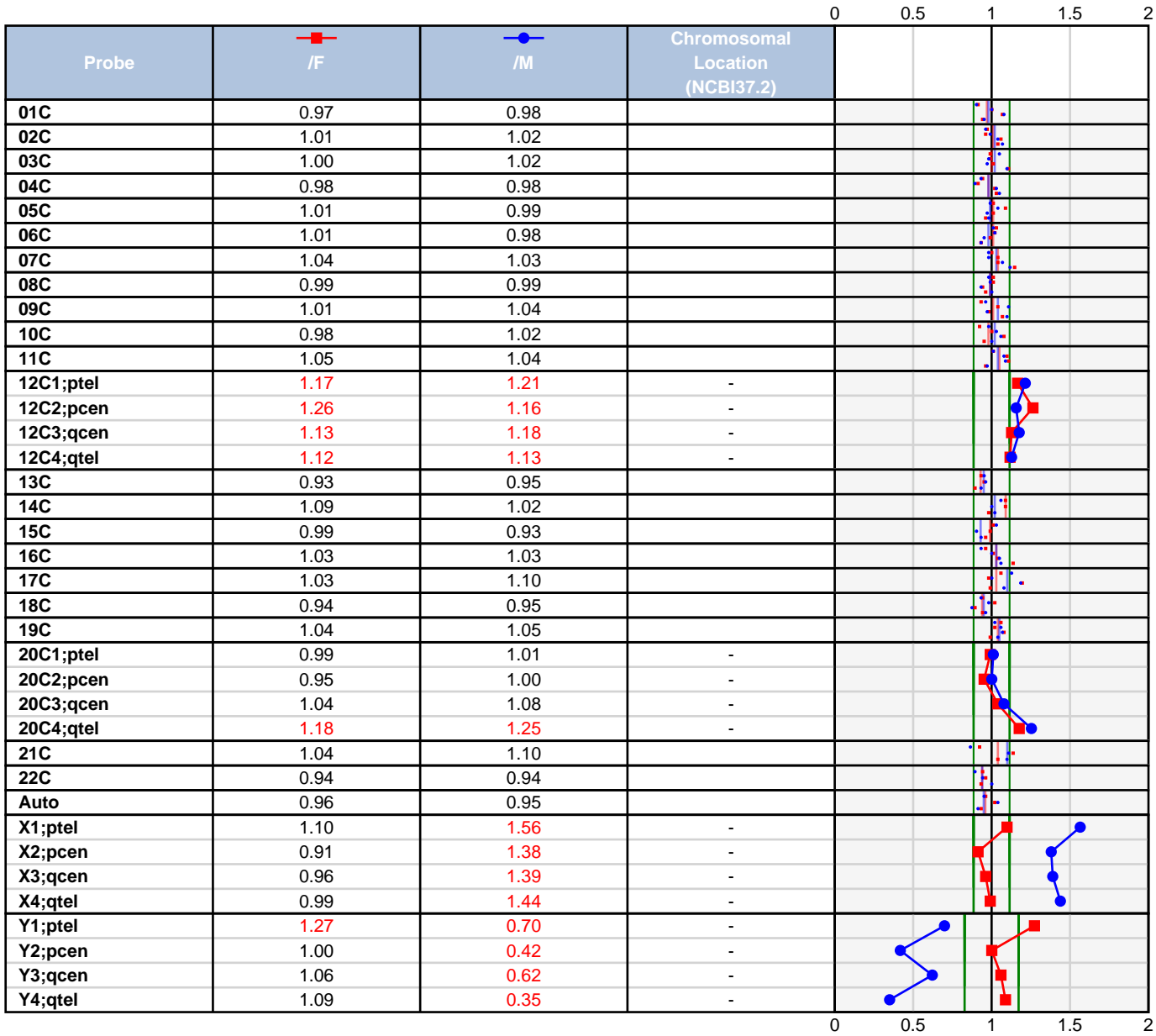

Supplement: Supplementary file 15 — Supplemental Figure 6. Karyotype analysis with array comparative genomic hybridization technique. Results showed normal karyotype of SNPH-/- embryonic stem cell line, clone 5A, in RC17 WT background. [file 10020_2025_1319_MOESM15_ESM.pdf]

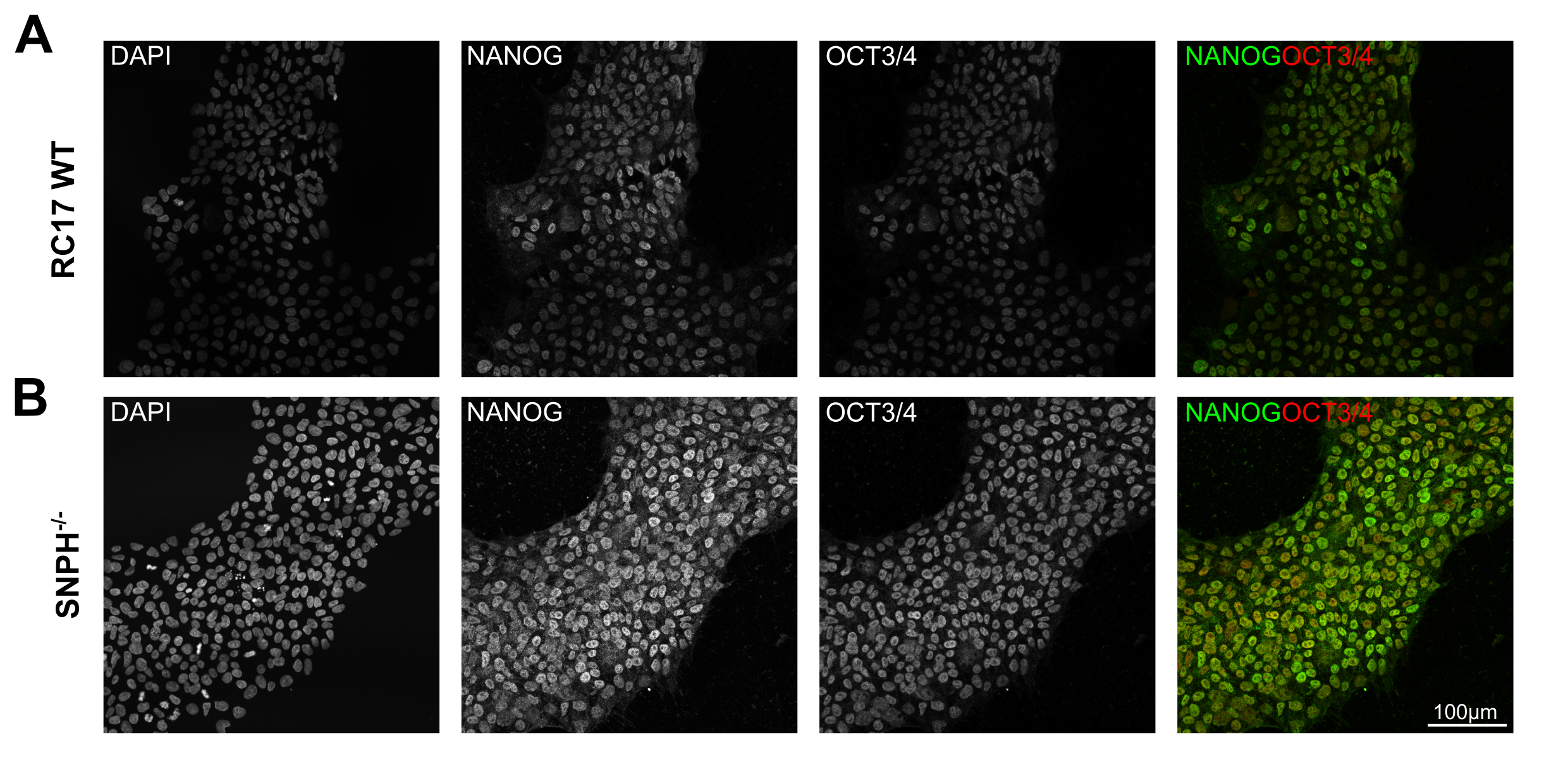

Supplement: Supplementary file 16 — Supplemental Figure 7. Pluripotency immunocytochemistry of WT (A) and SNPH-/- (B) embryonic stem cell lines. Immunocytochemistry of NANOG and OCT3/4 staining, two pluripotency marker, in embryonic stem cells, at passage 35 and 47 for WT and SNPH-/-, respectively. Scale bar: 100µm. [file 10020_2025_1319_MOESM16_ESM.tiff]

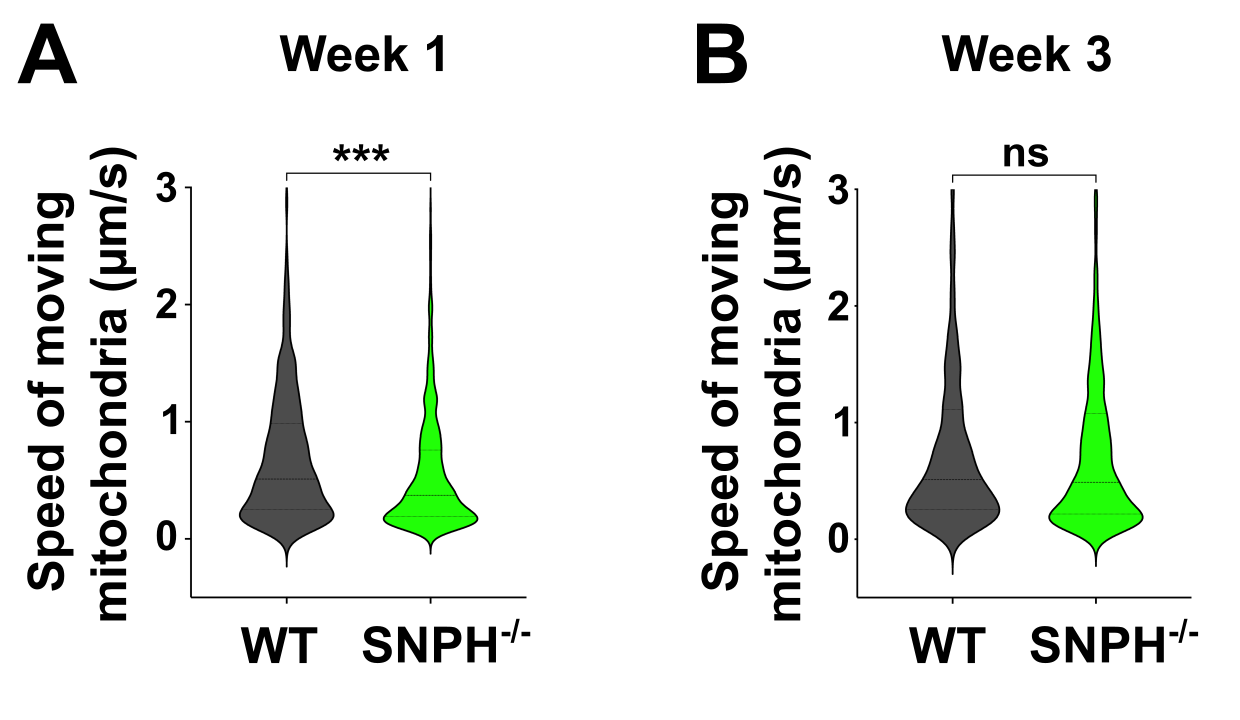

Supplement: Supplementary file 17 — Supplemental Figure 8. Speed of moving mitochondria. Speed of moving mitochondria (µm/s) was significantly different between WT and SNPH-/- MNs of (A) one-week-old motor neurons, but unaltered at (B) three-weeks. Each datapoint represents one mitochondrion. Mann-Whitney test, one week: mean WT=0.69mm/s, mean SNPH-/-=0.56mm/s, p=0.0002, N=6, n=245-502; three weeks: mean WT=1.13mm/s, mean SNPH-/-=0.95mm/s, p=0.36, N=6, n=232-614. [file 10020_2025_1319_MOESM17_ESM.tiff]

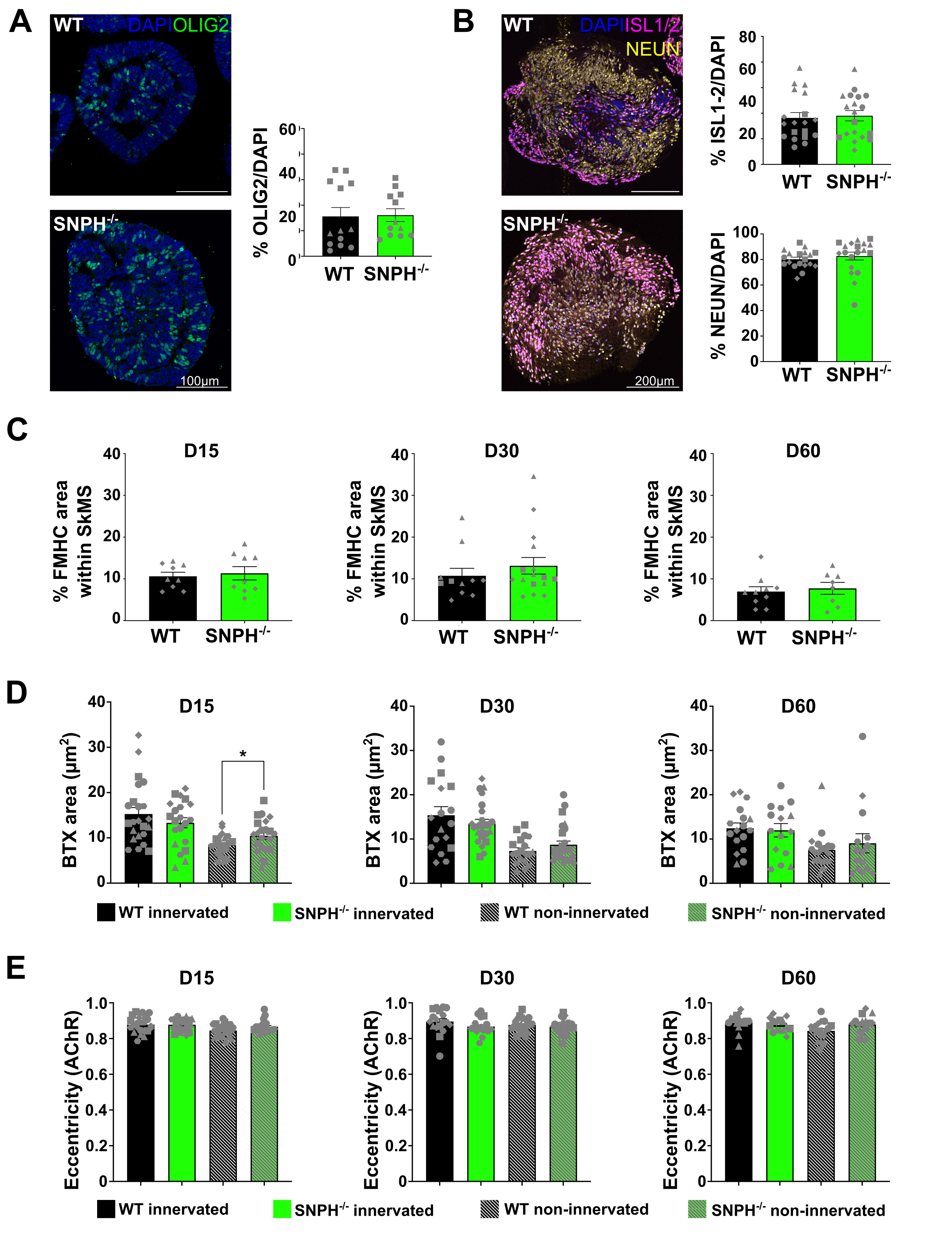

Supplement: Supplementary file 18 — Supplemental Figure 9. SCO differentiation, SkM area and AChR size were not altered upon SNPH loss. A: Loss of SNPH did not lead to altered numbers of OLIG2+ neuronal progenitor. DAPI: nuclei, OLIG2: motor neuron progenitor. Scale bar: 100 µm. Mann-Whitney test, WT=31.2%, SNPH-/-=32.2%, p=ns, N=3, n=12-13. Each datapoint represents one SCO. Different shapes represent different N. B: Loss of SNPH did not lead to altered numbers of ISL1/2+ or NEUN+ neurons. An eccentricity of 0 represents a spherical shape, while eccentricity of 1 represents a linear shape. DAPI: nuclei, ISL1/2: motor neuron marker, NEUN: pan-neuronal marker. Scale bar: 200µm. Mann-Whitney test, ISL1/2: WT=36.3%, SNPH-/-=38%, NEUN: WT=80.2%, SNPH-/-=82.7%, p=ns, N=4, n=19. Each datapoint represents one SCO. Different shapes represent different N. C: The percentage of FMHC+ area within SkMS of NMAss was not altered. Unpaired, parametric t-test, p=ns (D15, D60), Mann-Whitney test, D15 mean WT=10.6%, mean SNPH-/-=11.3%, D30 mean WT=10.7%, mean SNPH-/-=13.1%, D60 mean WT=7.0%, mean SNPH-/-=7.7%, p=ns (D30), N=2-3, n=8-16. Each datapoint represents one NMAss. Different shapes represent different N. Data presented as mean ± SEM. D: Quantification of post-synaptic BTX+ area of innervated or non-innervated AChR cluster showed no significant difference between genotypes with the exception of D15 non-innervated AChRs. Welch’s t-test or Mann-Whitney test comparing innervated or non-innervated AChR cluster between genotypes, NFH+/BTX+ AChR cluster mean D15: WT= 15.3µm2, SNPH-/-=13.3µm2, p=0.67, mean D30: WT=15.4µm2, SNPH-/-=13.5µm2, p=0.39, mean D60: WT=12.4µm2, SNPH-/-=12µm2, p=0.82, NFH-/BTX+ AChR cluster mean D15: WT=8.5µm2, SNPH-/=10.5µm2, p=0.04, mean D30: WT=7.3µm2, SNPH-/-=8.7µm2, p=0.37, mean D60: WT=7.5µm2, SNPH-/-=9µm2, p=0.89, N=4-5, n=15-26. Each datapoint represents one NMAss. Different shapes represent different N. Data presented as mean ± SEM. E: Eccentricity of BTX+ AChR cluster was not altered be [file 10020_2025_1319_MOESM18_ESM.tiff]
